# Firefly: A Low-Resolution Thermal Trigger for Gated Sensor Fusion in Human–Robot Collaborative Manufacturing

**DOI:** 10.3390/s26206482

**Published:** 2026-10-13

**Authors:** Olga Podkorytova, Mingcheng Yang, Yancheng Chen, Maxwell Mamishev, Eric Jiang, Zheng Liu, Xueyicheng Xu, Sep Makhsous

**Affiliations:** 1 Sensors, Energy, and Automation Laboratory, University of Washington, Seattle, WA 98195, USA; podkorytovaoe@gmail.com (O.P.); myang24@uw.edu (M.Y.); ryanch77@uw.edu (Y.C.); maxm7@illinois.edu (M.M.); ericjiang799@gmail.com (E.J.); zheng726@uw.edu (Z.L.); xueyix@uw.edu (X.X.); 2 Department of Electrical and Computer Engineering, University of Washington, Seattle, WA 98195, USA; 3 Department of Electrical and Computer Engineering, University of Illinois Urbana-Champaign, Champaign, IL 61820, USA

**Keywords:** infrared image sensors, LiDAR sensors, sensor fusion, computational efficiency, intelligent manufacturing, sensor systems and applications

## Abstract

**Highlights:**

Functional and reliable collision-avoidance systems are required to create safe and efficient production systems within Industry 4.0 intelligent manufacturing environments. However, only about one-third of employers in high-hazard industries use collision-detection technology. The manufacturing floor represents a dynamic and complex work environment. Therefore, no single system currently available can meet all the requirements of the manufacturing floor. Sensor fusion restores coverage by providing data from multiple sensors. However, each additional always-on layer adds both power consumption and cost. In this paper, we examine the potential use of a sensor-fusion platform that pairs an always-on, low-power thermal trigger with a gated LiDAR. We also introduce Gated-Fusion Efficiency (GFE), a figure of merit for the value of the measured trigger to the stack’s power budget and system complexity.

**What are the main findings?**

- Low-resolution thermal human detection is capable of detecting people at industrial vehicle ranges using an 8 × 8 thermopile sensor array. This can differentiate a standing person from a body-shaped passive thermal mass based on a single threshold that is fitted once and then held fixed. The mean F1 for this system is 0.875 over the distance range from 1.2 to 7 m, and 0.868 for a standing person in a held-out validation experiment on the floor of an operating factory. However, the usable distance range is determined by the environmental conditions where the system is deployed. In thermally uniform environments, F1 is nearly perfect at every distance. In cluttered environments, however, F1 collapses beyond 5 m.
- The single-frame, two-gate method uses the background temperature contrast only to localize the warm cluster, and scores discrimination on raw temperatures. This method has one threshold that is used in every environment, and it does not use any training data or temporal windows.
- Two components comprise the efficiency-assessment framework for embedded triggered sensing: sensor characterization and Gated-Fusion Efficiency. These components are demonstrated end-to-end on Firefly. The average power saving of the stack Firefly gates was determined from its measured wake rates. At a 5% occupancy rate, the average power saving was found to be 1.29× at node scope, with no task quality given up.

**What are the implications of the main findings?**

- Low-resolution thermal sensing fills a gap in industrial collision-avoidance stacks that no existing modality covers. It finds stationary workers who are missed by passive infrared (PIR) sensors, without depending on light as cameras do. It also solves the problem of LiDAR being unable to distinguish a human from clutter of similar shape.
- The detection range of a thermopile is dependent upon the deployment scene, not the sensor itself. Using the per-pixel field of view and the target width, deployers may determine the distance at or beyond which the range will be scene-dependent. At that point, the range should be validated in the actual deployment environment.
- The assessment framework applies to any trigger–stage pairing prior to construction. Gated-Fusion Efficiency converts measured detection rates and false-alarm rates into the average power saving of the gated stack. This average power saving is limited by both the relative power of the gated stage and the reciprocal of the false-alarm rate of the trigger. Therefore, more expensive gated stages demand cleaner triggers.

**Abstract:**

Automated guided vehicles and mobile robots are present on many Intelligent Manufacturing factory floors. Approximately 7.0 million U.S. workers also work on those same floors. During 2011 through 2017, forklifts caused 614 worker fatalities. Currently, no commercially available collision-avoidance sensor can manage the complexity associated with working within a plant. Therefore, fusing multiple modalities allows a system designer to gate the more expensive ones behind some minimal signal that indicates all systems should be active. Such minimal signals are expected to include body heat that even stationary personnel would have difficulty suppressing. In addition, the signal-capturing sensors ought to operate at milliwatt levels of power while keeping workers’ privacy. One type of device that has been found suitable for these requirements is a coarse thermopile array. Such a device can be fused with a rangefinder that determines distance to form a gated system. Yet the measurement capability frontier of the thermopile arrays in practical scenarios has not been explored. In addition, there is currently no figure of merit to quantify the trade-ff between induced errors and system-level power saving. In order to address these gaps, we built the fusion system, Firefly, together with a training-free detector and tested them against a human and a mannequin 1.2 to 7 m away in four thermally distinct environments, one of which is the floor of an operating factory. We found that the thermal array’s detection range varies with the environment but is predictable in a thermally uniform environment. Based on these experiments, we also proposed the Gated-Fusion Efficiency (GFE), a figure of merit that converts a trigger’s error rates into system-level power savings. Together, the capability boundary and the GFE help guide sensor-fusion system design in industrial manufacturing toward safer working environments.

## 1. Introduction

Industrial automation is one of the main technologies behind intelligent manufacturing. Roughly 7.0 million American warehouse and factory workers now work alongside automated guided vehicles and autonomous mobile robots [1,2,3,4]. Contact accidents between these vehicles and workers stay frequent and severe. Occupational Safety and Health Administration (OSHA) estimates 35,000 serious forklift-related injuries every year. It estimates another 62,000 non-serious forklift-related injuries. Between 2011 and 2017, 614 workers have died due to forklift-related incidents [5,6] with most fatalities resulting from pedestrians being struck by the forklifts. This is the event a collision-avoidance sensor exists to prevent [6].

A commercial marketplace for collision-avoidance sensing systems has been established in response to these hazards [7,8,9]. Advanced commercial products are available. The manufacturing environment remains one of the hardest application scenarios for them, for three reasons. First, a manufacturing floor is a three-dimensional space. Potential hazard areas include overhead racking and elevated levels (mezzanines), as well as heavily loaded items being moved. Automotive collision avoidance systems generally assume a relatively flat road surface. Second, there is significant variability among production environments based upon product type. For example, a steel processing plant will have very little commonality when compared to a toy factory regarding overall environmental characteristics. Therefore, solutions designed for one production location will likely be less effective than desired at other locations [10]. Finally, the production floor is populated by many objects at a human scale, including but not limited to racking legs, bollards, and wrapped pallets, that a collision-avoidance sensor must identify and determine if they represent a person. Adoption figures show these difficulties. Across high-hazard industries, 87 percent of surveyed employers consider collision-detection technology applicable to their hazards. Only approximately one-third actually use it. Cost continues to be cited as the leading deterrent for purchasing this type of equipment [11].

The difference between applicability and usage comes from technological limitations of each commercially used sensor technology. Every commercially available and widely applied presence detection method can fail in at least one common application on a factory floor. PIR-based occupancy sensors are the lowest-cost and most widely installed detection solutions for people. These sensors operate by detecting changes in the amount of infrared radiation received by them [12,13]. As such, a person who is simply standing at the end of an aisle will produce no response. Vehicles need to identify this particular scenario. Sensors using ultrasonic and/or radar technologies can detect stationary objects. However, they provide only information about how far away something is from them and do not indicate what type of object is being detected [14,15]. Therefore, if both a wrapped pallet and a person were located in the sensor’s field of view, both would likely trigger alarms. Since vehicles cannot determine whether the detected obstacle was a person or a wrapped pallet, vehicles will have to treat all obstacles as if they were a person. If they fail to do so, then there is a risk of missing the actual person. Safety-rated laser scanners have solved the problem of detecting the distance of objects regardless of lighting conditions. However, these systems come with higher costs than other commercially available systems and only determine the geometry of sensed objects. For example, a laser scanner may see both a stationary worker and a stationary bollard as the same object because they are of similar size [15,16,17]. Industrial camera systems have the ability to identify people, as opposed to objects such as bollards. However, they are dependent upon adequate illumination. Industrial interior environments provide glare, dust, and often night-shift working conditions [18,19]. Because of this, illumination is a major limitation of industrial camera systems. Camera detection requires a pipeline of 8 to 40 giga floating-point operations (GFLOP). These run on a Jetson-class device that draws several watts [20,21]. Cameras also record faces and identities. Privacy regulations can conflict with camera-based person recognition in an industrial environment [22,23]. Worn on a worker’s body, ultra-wideband (UWB) or radio-frequency identification (RFID) tags do not perceive the surrounding environment. The only workers that are protected are those wearing these types of tags. All movements made by a worker wearing the tag are tracked [24,25]. Untagged visitors or contractors cannot be identified in the system. Each approach has benefits but introduces a failure of its own. There is no single solution that can recognize a stationary person, distinguish between a person and person-sized clutter, and avoid recording an individual’s identity. Therefore, industrial practices rely on combining multiple different sensor technologies into a singular perception stack [4,14]. Adding additional layers to the perception stack results in increased power consumption, increased computing requirements and increased costs [20,21].

Solving these deficiencies will require fusing sensors. Sensor fusion combines different sensing modes that complement each other to address each other’s shortcomings [26]. The problem is what combination provides the necessary coverage at a deployable power and cost. By deployment, we mean onto all vehicles, not just a pilot vehicle. There are two types of devices that can naturally combine together since individually they have limitations. First is the low-resolution thermopile array. Because heat is the only signal that a stationary worker cannot mask, there are thermal channels present in some existing vehicle sensor fusion packages [27,28,29]. High-resolution full thermal cameras would increase the overall cost of the sensors. In addition, high-resolution thermal images have been used for identifying people [30]. Low-resolution thermopiles can be bought at commodity prices. They avoid the cost and the potential to identify an individual using thermal images. Low-resolution thermopiles provide three advantages. They use milliwatts of power instead of watts. Their outputs are small enough to be processed by a microcontroller. They resolve neither faces nor gaits. Their resolutions are below the levels at which thermal identification has been demonstrated [30,31,32,33]. However, low-resolution thermopiles do not measure distance, and they deliver only a coarse thermal image. The second type of device is the single-point LiDAR rangefinder, which returns precise distances at milliwatt power but does not include any information regarding the target object being sensed. In conjunction, each device covers the other’s blind side. The thermopile reports that a person is present in its sensing area. The rangefinder then provides information on how far that individual is from it. The portion of the task assigned to the thermopile has no experimental validation in the deployment configuration. Thermopile human detection is validated overhead. Ceiling-mounted arrays count room occupants with accuracy above 95% [34,35]. Vehicle-mounted sensors view their surroundings horizontally. Their range will be defined by the stopping distance. As humans move further away from the sensor, their bodies occupy fewer pixels. Warm clutter may also appear at a human scale [34,36]. The detection issue now becomes determining if a warm region seen by the sensor represents a human. This essentially describes the bollard problem again, but using heat instead of visual imagery. To date, no research has determined if a thermally based, 8 × 8-class array can perform human versus non-human discrimination beyond 3 m. The test case is a passive object that creates a body-shaped warm cluster. Testing should occur across multiple thermally distinct environments.

Fused stacks create an additional engineering challenge with regard to energy. A low size, weight, and power (SWaP) platform has a limited amount of power available. All modalities that are running continuously add their total consumption to the node. The total amount of power consumed will be determined by the most expensive modalities operating continuously. On a battery-powered vehicle or a retrofit node, that budget decides whether the system ships [37]. The standard approach to this issue is gate-based. One low-power sensor always stays on. This cheaper, lower-power sensor will wake up the rest of the more costly stages only if there is some necessity for the remainder of the circuitry to be active. Gating is established practice. It is standardized in cellular wake-up receivers [38]. For over twenty years, researchers have studied wake-up sensing and identified use of gate-based architectures as one of the primary approaches [39,40]. These architectures are used in various systems, including infrared front-end activated camera nodes [41]. The cheap, low-power sensor will earn its position as the always-on trigger. The expensive stages of the system will only be powered up when the always-on sensor detects something that requires additional energy consumption. Today, selecting the always-on sensor (or the trigger) for a gate-based architecture has no system-level justification. Wake-up sensors can be characterized using three metrics: power, detection accuracy, and false positive rate, with each metric being reported separately [39]. Two of these metrics will determine how long the gated stages operate. The first is how often the trigger fires when a person is present. The second is how often it fires when no one is present. No existing figure of merit translates these two metrics into the total power saving for the entire device stack. The total power saving is what designers require.

**Figure 1:**
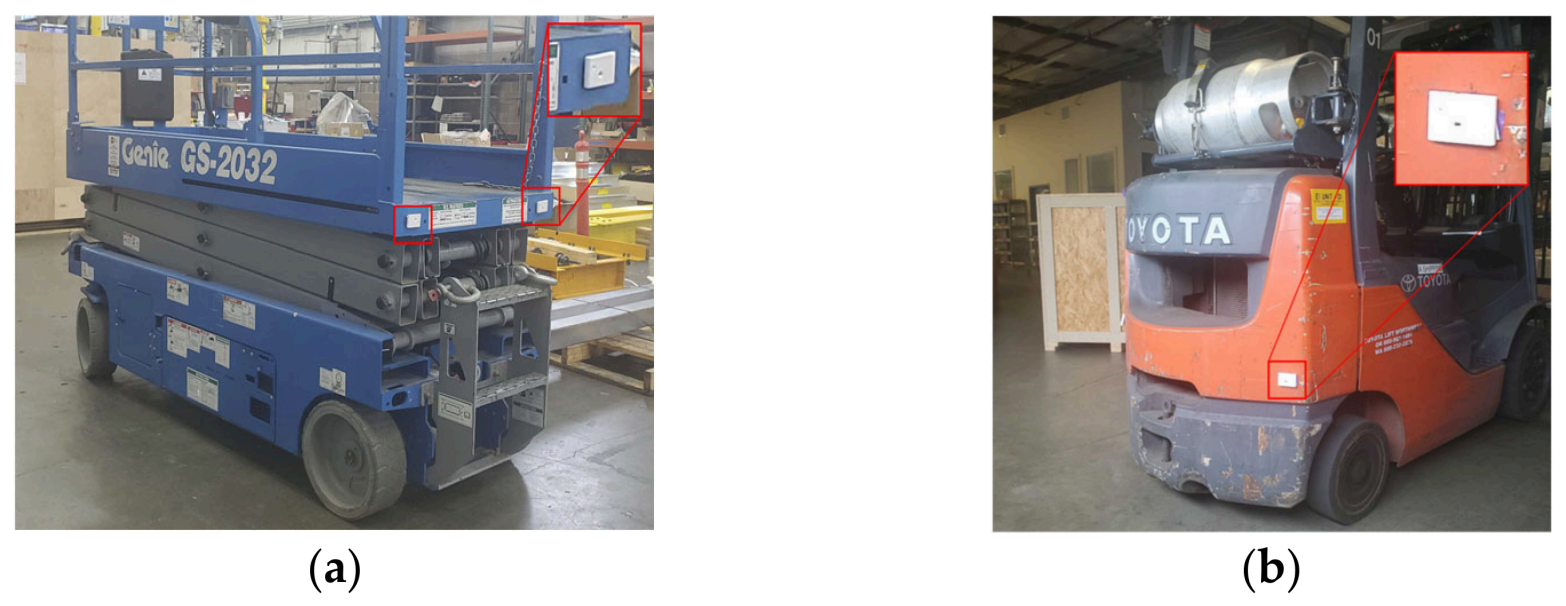
Firefly nodes mounted on two industrial vehicle classes, showing candidate mounting positions: (**a**) scissor lift with two nodes on the platform base; (**b**) counterbalanced forklift with one node on the rear counterweight. Zoomed-in insets show the nodes. All trials in this paper used a stationary tripod (Section 4.1).

Firefly is a multi-sensor fusion-based system for Intelligent Manufacturing workplace safety, specifically, collision avoidance (Figure 1). Firefly uses an always-on, low-resolution thermopile (thermal) detector as a trigger and a gated single-point LiDAR range finder. Firefly uses a single-frame, training-free detection framework. In this paper, we address both of these open questions. We characterize the performance of the thermal trigger over four different environments, including an operating factory environment, at six different ranges. We develop a figure of merit for the value of any measured trigger to the stack that it gates. The main contributions of this paper are as follows:

- The initial operational envelope for detecting humans with 8 × 8 thermopiles at the distance of an industrial vehicle. The negative example for this application would be a warm object of a similar shape to the human body. Detection of a stationary person against such an object is nearly perfect in controlled environments up to 7 m. At close range, F1 = 1.000. In both indoor and outdoor scenes, the sensor will continue to provide reliable detection even in cluttered environments up to a maximum of 5 m. However, at greater ranges than this, it will fail only in cases where there is a high degree of thermal complexity present. Where the sensor fails as a function of environmental complexity is set by three parameters: the per-pixel angle of view given in the product sheet, the dimensions of the target (the human body), and the cluster size that the detector requires. Therefore, a deployer can determine where any given thermopile-based sensor becomes dependent on its environment, at any resolution.
- A two-gate, single-frame detection framework that separates the two jobs which background subtraction usually does at once. The first stage uses the contrast image only to locate a warm cluster. The second stage computes a discrimination score from raw temperature values. This makes it so that the second gate’s decision-making process is performed on data that were never modified or processed by the first gate. This eliminates the self-reinforcing confirmation bias inherent in the standard residual-scored pipeline. In addition, it maintains the system as being completely free of any need for either training data or temporal windows. A safety-critical trigger requires both properties.
- Gated-Fusion Efficiency (GFE) is a figure of merit for a gated system. It uses the two measured detection rates for a given trigger as inputs. These detection rates are used to determine the average amount of power saved when using a gated stage versus a non-gated stage. This determination is done at a declared system scope. GFE can be utilized with the measured operating points of Firefly. Therefore, it quantitatively measures the power saving achieved by the node at realistic occupancy levels. A builder can take two design rules from GFE into any new system. The first rule has to do with the power of the gated stage. The maximum amount of savings that will be possible is limited by the relative power of the gated stage. The second rule has to do with the false-alarm rate of the trigger. A trigger cannot save more than the inverse of its false-alarm rate. Both of these rules are true for all gated sensing systems, not just Firefly.

The rest of this paper will be structured in the following manner. Section 2 contains an overview of currently deployed collision-avoidance sensing and low-resolution thermopile human detection. In addition to that, it provides a review of single-frame detection techniques and triggered sensing along with their efficiency metrics. Finally, it explains the gaps that exist. In Section 3, the sensor node, its coverage geometry, the two-gate detection algorithm, and the GFE formulation are all described. In Section 4, an explanation of how the experiments were designed and run is given. In Section 5, the results for SNR separation, the F1 envelope, and the GFE instantiation are reported. Section 6 provides an analysis of the boundaries placed on gating designs, the resulting selection rules based upon those boundaries, the latency and stopping distance of the node, and the limitations. Finally, Section 7 concludes the paper.

## 2. Background and Related Work

### 2.1. Industrial Collision-Avoidance Sensing

Five sensor families account for the majority of installed collision-avoidance sensors. These families include: (1) passive infrared sensors, (2) ultrasonic and radar ranger sensors, (3) safety-rated laser scanners, (4) cameras, and (5) tags worn by workers. Three constraints limit each family’s use in a shared manufacturing environment. First, a stationary individual must be recognized by the system. Second, an individual must be distinguished from other objects of similar size, that is, from clutter. Third, the identity of an individual must remain uncaptured by the system. This section reviews why none of the sensor families are able to satisfy all three requirements. Next, we will look at the existing literature on low-resolution thermopile arrays and single-point LiDAR rangefinders. Then, we will also review the literature concerning single-frame detection methods which are required by the trigger function. After that, we review the literature concerning triggered sensing in relation to trigger performance and stack power. The conclusion from surveys of human-detection sensing for collaborative and mobile robots is that there does not exist a singular modality capable of covering the full operating envelope. These same surveys conclude that fused multi-sensor stacks currently dominate the practice [4,14].

A passive infrared sensor responds solely based on the change in thermal radiation over time within its area of detection. The sensor will not detect a subject that remains at a consistent thermal level [13]. A worker who has stopped moving will fall under the detection threshold for a PIR-only stack [13]. This would be an unacceptable failure with regards to collision avoidance. All stationary people in a vehicle path must be detected [4,14,24,42].

Radar and ultrasonic range finders in industrial vehicles are both in the near field of these vehicles. Reviews of external sensors for detecting people in collaborative environments describe ultrasonic devices as small and cheap. They have been reviewed as usable only at short distances and are deployed almost exclusively in combination with other sensors [14]. Radar extends the ranging capability of ultrasonics. Like ultrasound it will return presence and distance without classifying the object [14]. Neither device will discriminate between human and object. The literature reports no rate of discrimination for either device [14]. Therefore, a wrapped pallet, cart, or stacked carton within the beam will produce the same alarm as a person. A range-finding-only vehicle must either stop on every return or suppress alarms, including true ones. Radar that senses vital signs is the exception. A 60 GHz millimeter-wave (mmWave) radar can detect and count people, including people who are not moving, by sensing their vital signs and minute motions [43]. This capability carries a power cost. A 24 GHz radar presence module averages 80 mA at 5 V [44], and a 60 GHz mmWave sensor with on-chip signal processing averages 1.19 W to 1.75 W [45]. These figures are 27 to 117 times the 15 mW drawn by a low-resolution thermopile array [33]. At this power, such a radar suits a gated stage better than an always-on trigger layer.

Safety-rated laser scanners and LiDAR form the certified geometric layer of vehicle safety. The primary barrier for their adoption is their cost in terms of overall system price, as identified by the deployment literature [15]. LiDAR captures the direct three-dimensional geometry based on returning light independently of the ambient lighting conditions. However, it contains no thermal or material information [16,46]. Therefore, discrimination between a stationary individual and a human-sized structure will be solely determined by its shape. A human-sized structure can have the exact same general shape as a person. Structured laser scanning provides higher density geometry compared to a scanning rangefinder; however, it requires a patterned illumination source and per-scan processing [47]. Additionally, detection decreases with distance since returns become increasingly sparse on human-sized objects. The crane-mounted literature indicates that the constraints imposed due to decreasing returns with increasing distance are the main limiting factor at industrial working distances [48]. Because LiDAR does not capture facial identities, LiDAR has been described as privacy-preserving [17,49,50,51]. However, gait recognition can determine an individual’s identity from a cloud of points. On a 1050-subject benchmark, gait recognition achieved a rank-one accuracy of 86.8% and reached 91.8% accuracy on walking sequences under normal walking conditions [52]. For both red–green–blue (RGB) cameras and high-resolution LiDAR, the only way to ensure there is no risk of identification is to reduce sensor resolution to levels that do not support it. This is established through the hardware and not through a software layer.

Camera-based detector systems are designed to contain rich semantic data per frame. A well-developed pipeline exists around these systems [53]. Three limitations follow from these systems’ structure. Cameras operate based upon light. However, they will degrade when subjected to the shadows, glare, or low-light conditions that occur in industrial settings and night-shift yards. As a result, recent forklift and advanced driver-assistance system (ADAS) studies fuse RGB streams with thermal channels [27,28,29] as opposed to using an RGB camera by itself. High computational costs exist because edge-deployments of pedestrian detectors utilize lightweight You Only Look Once (YOLO) variants which fall within the 8 to 40 GFLOP range. Achieving real-time processing speeds requires Jetson-level devices operating at several watts [20,21], thus creating a minimum for both platform price and power. Due to their inherent nature, RGB cameras capture faces, gait, and clothing, which creates a recognizable identity [22,54]. The privacy implications associated with this capability may be addressed through software-level obfuscations applied to the original video stream [54,55]. This mitigation must be validated per deployment. This type of solution is not a structural privacy guarantor and is subject to increasing scrutiny from privacy regulation [23].

The worker-worn tags method approaches this issue from the person’s perspective. The localization of a tag worn by each employee occurs through ultra-wideband and RFID (radio frequency identification) systems. Once both the tag and vehicle have converged at a common point in space, an alert will be raised. Each published design for this technology has been influenced by the overall cost of deploying it, as well as the specific site conditions, and privacy and data protection requirements [24,25]. The structure created for this technology also creates its own limitations. Only employees who wear tags are protected. All movements of tagged employees will be recorded. An untagged visitor or contractor would remain completely undetectable by the system [24,25]. The functionality of detecting is located on the individual wearing the tag instead of within the scene.

Every family leaves at least one of the three requirements unmet or meets them only at a power that rules out always-on use. Industry responds to these unmet needs by combining some or all of the above-mentioned families into a perception stack. Surveys identify fusion as the predominant architectural design [4,14]. Deployed collision avoidance systems pair LiDAR with cameras in real time [56]. Each additional sensor is constantly running. Therefore, every new addition to a vehicle’s stack increases the amount of power required, increases the amount of processing it requires, and increases the cost to build [20,21]. Such a stack still lacks an affordable always-on trigger layer covering the cases the other modalities miss. In this paper, we explore whether low-resolution thermal sensing combined with single-point ranging will meet that need.

### 2.2. Low-Resolution Thermopile Arrays for Human Detection

As a trigger, our sensor must (1) detect people, even when they are not moving, (2) not capture enough detail to identify them, and (3) operate within a milliwatt power budget for continuous operation. The first of these is closest to occupancy sensing in the literature, though the two are not identical. For example, one study [57] interpolated data from an 8 × 8 AMG8833 array and used background suppression and adaptive thresholding to sense occupancy. For activity recognition, an 8 × 8 sensor has been used with over 98% accuracy [35], and a 32 × 32 sensor has been used to detect multiple people with over 98% mean average precision in the presence of challenges including warm air streams, solar radiation, and cold objects [34]. In terms of preserving privacy, human identification using thermal imaging was shown to be feasible with camera imagery of six enrolled individuals down-sampled to a resolution of 16 × 12 [30]. Sensors from the 64-pixel class fall short of that resolution. These examples establish what can be accomplished with similar devices, but do not answer what happens with our geometry.

A vehicle-mounted sensor views the floor horizontally, over a range determined by the stopping distance. Almost every previous validation has instead mounted the sensor overhead, viewing the subject from above against a thermally uniform floor [58,59,60]. Placing the sensor in this manner provides the greatest possible thermal cross-sectional area of a person [58] while limiting the range of distances through which an object can be viewed [59]. The overhead placement also presents confounding variables as heat anomalies, rather than as objects with human-like dimensions and shapes. In contrast, horizontal positioning removes each of these advantages. Because the body takes up fewer pixels as distance increases, and because warm clutter can take on both the same size and apparent temperature characteristics as a human [58,59], the discrimination problem shifts from identifying where there are warm areas to determining if the identified warm area represents a human [58,59]. No research has investigated how much discrimination exists in the 8 × 8 class under horizontal viewing beyond 3 m. The most similar quantifiable comparison would be from the horizontally oriented system, TADAR [61]. TADAR is implemented with a 32 × 24 array, twelve times the pixel count of an 8 × 8 array. On average, TADAR’s F1-score for multi-user detection was 0.888. As users occupy fewer pixels, the ranging error increases. This error was measured at 20.1 cm within 3 m. The ranging error then increased to 39.4 cm over the 3 to 4.5 m band [61].

A fixed deployment budget dictates that we can only use the 8 × 8 tier. With milliwatts of power and commodity costs, we are able to provide an always-on layer on each vehicle. Prior work has used higher resolutions, such as 32 × 24 or 32 × 32, as a way to address coarse spatial granularity in thermal sensing. Higher resolution allows us to better separate multiple people who appear as one thermal blob when using lower resolutions [58,62]. It improves multi-person separation and activity recognition [58]. It addresses crowding, but it does not help with discrimination between a human and a passive object. It is also not available within the cost and power limits of the 8 × 8 tier.

In terms of a similar previous test configuration, Dvorak et al. [63] deployed a thermal array about waist-high in front of both sitting and standing human test subjects out to 3 m. Their setup simulated proximity to autonomous agricultural machinery, and they found a consistent ability to detect heated models out to that distance [63]. For their testing purposes, Dvorak et al. used as their negative test model an unheated mannequin which equilibrates to ambient and produces no detectable thermal cluster. Therefore, the discrimination task for them reduced simply to determining whether a thermal signature was present or not [63]. In this work, a much more difficult control was chosen. A passive mass was used instead, which absorbs ambient heat and presents a body-shaped thermal cluster. To date, there are no thermopile studies that have tested the 8 × 8 class against that case. Dvorak et al. worked at the pixel level. They also recommended that deployments should ensure that the observable area per pixel matches the expected size of heated regions [63]. This paper formalizes that coverage-geometry account.

The single-point LiDAR rangefinder is the ranging candidate. LiDAR rangefinders are a relatively low-cost, commodity time-of-flight (ToF) device which provide a single calibrated distance measurement at low power, in the milliwatt range. They can be found in many different types of robots for the purpose of detecting obstacles [64,65]. Although LiDAR rangefinders do not provide any information about what type of object is detected, and there is no object classification or identification, the fact that they can measure how far away something is makes them useful in many applications [66,67]. There are multizone versions of this technology that have been used for presence detection on their own [68]. The single-zone tier trades that capability for the lowest cost and power in the class.

Neither one of these sensors individually would meet this collision avoidance requirement. The thermopile reports that a person is present but does not report the distance to that person. Therefore, the thermopile could never be used as input for making a stopping decision. The rangefinder will report the distance to all objects regardless of whether they are people or person-sized objects. So, while the rangefinder can report how far away an object is, it carries no information about what type of object it is. When paired together, these two are complementary. Experimental support in the deployment geometry is missing for the thermal half of the pairing.

Three structural design parameters distinguish this research from previous work. These are the orientation of the sensor’s line-of-sight (viewing axis), the distance range over which the sensors operate, and how the negative class is defined. Overhead studies avoid the discrimination problem through geometries that keep the detected human large in the frame while making confounders rare [58,59,62]. Horizontal, high-resolution arrays are denser by an order of magnitude compared with the 8 × 8 array [61]. The closest 8 × 8 horizontal array study has a stopping point at 3 m, tested against an unheated form which produced no cluster [63]. No prior 8 × 8 work has operated under horizontal viewing at distances greater than 3 m against a passive object that presents a body-shaped warm cluster, tested across a variety of thermal environments.

### 2.3. Single-Frame Detection Methods on Low-Resolution Thermal Arrays

Detection on an 8 × 8 pixel array mounted on a vehicle starts by making a determination at each frame as to which pixels have a higher temperature than their surrounding area [58,69]. A reference point for ambient conditions is required in order to make this comparison. Background subtraction fulfills this role as a source of reference information [58,70,71]. Systems using indoor thermopiles produce estimates of relatively slow changing fields representative of the ambient temperatures of rooms. These are used to subtract out the ambient field leaving only newly formed or anomalously warm regions [58,71]. The ambient field is frequently estimated alongside a Kalman filter, or some form of adaptive method for updating the estimate to account for heating, ventilation, and air-conditioning (HVAC) drift and other ambient change [58]. The same type of processing occurs when attempting to separate a person’s thermal footprint from its surrounding environment in order to estimate distances within physical-distancing applications [72]. In all of this work, background subtraction serves only as the reference against which spatial detection is performed [58,71].

A reasonable follow-up would be to threshold the background-subtracted images to determine what portion of the image is part of the desired target. This is also the point at which low-resolution arrays deviate from traditional thermal imaging. Histogram-based thresholding techniques like Otsu and their related entropy-based versions rely on a filled, approximately bimodal distribution of intensities in the histograms [69], which a 64-pixel grid does not provide. Monte Carlo studies of Otsu segmentation have shown that stable operation depends upon two conditions: sufficient intensity difference between objects and backgrounds, and sufficient area of the object relative to the total image area [73]. Entropy-based thresholding tightens the second condition further, requiring that the object fills a substantial portion of the image [74]. Therefore, when using an 8 × 8 array to observe a distant human target, neither of these conditions can be satisfied. The target covers only a few pixels. The resulting histograms are therefore sparse and often unimodal. As a result, the threshold value becomes unstable [73,74]. Local thresholding methods instead set each pixel’s threshold from the statistics of a surrounding window [75], and adaptive thresholding has been applied to occupancy sensing on 8 × 8 thermopile arrays [57]. These methods need a window larger than the target, so that the target does not set its own reference, yet small enough that the background inside it is uniform. On an 8 × 8 array, the two requirements conflict. A 1.75 m tall test subject spans about two pixels at 7 m but about 20 pixels at 1.2 m, so a 3 × 3 window is filled by a near target and left without local contrast, while a window large enough to contain the target covers at least half of the array and reduces to the global case. The threshold used in this work is also adapted in every frame, but its statistics come from the top row of the array, which lies above the subject, so the target cannot bias its own threshold. Spatial coherence criteria grow a contiguous group of adjacent pixels outward from the warmest pixel. In terms of robustness within this spatially coarse thermal image, they outperform histogram-based statistics. They exploit the one property that remains true about a real warm body, which is that it occupies neighboring pixels instead of dispersed ones.

In the discrimination step, after a candidate area has been identified, it will be decided if the warm area is a person or a passive source of heat. The most common method used to make this decision is based upon the signal-to-noise ratio (SNR) as described by [76]. There are several different SNR formulations that have been developed. The raw signal-to-noise, contrast-to-noise, and local signal-to-background ratios each describe a target intensity or contrast divided by a background standard deviation [76]. Small-target detection in infrared images uses the closely related signal-to-clutter ratio. This ratio is calculated from the raw image using two windows. One window is placed over the target area, and another window is placed over the background [77]. The authors of these studies have followed a consistent methodology in their research. The signal-to-noise ratio (SNR) has been calculated using the residuals from the background subtracted images as opposed to the raw image values [76,78]. By subtracting off the estimated background level, the variance associated with the background will be reduced, thus increasing the reported SNR. This increase in SNR is real. However, there is a cost that is associated with this approach. The same background estimate that identified where the target was located has also defined how the residuals were generated for use in calculating the SNR. Therefore, residuals associated with noise pixels sit near zero by construction. Residuals associated with cluster pixels are large by construction. Since both the discriminator and localizer have access to the same estimation history, when measuring the separation between the target and background, the data used to select the target will always show larger separations than data that did not select the target.

A second area of research has employed temporal information to help overcome the single-frame discriminability issue. Methods using persistence follow thermal blobs over frames and label static blobs, those lasting many frames, as clutter [79]. Learned detectors reach higher levels of discrimination accuracy on denser detector arrays [34]. Both types of methods have shown effectiveness, and both impose limitations which make them difficult to use by a collision avoidance system’s front end. Temporal methods require multiple frames and a transient start-up phase before they can make decisions [79]. Therefore, a vehicle moving towards the end of an aisle cannot wait for its frame window to become filled. To be used in a safety-critical application as a trigger, the safest option is to operate on a one-frame basis without any dependency on previous frames or the use of a learned model. Using this approach, the coupling between localization and discrimination is maximized since temporal smoothing, which would otherwise dilute the relationship, does not occur. Prior single-frame processing techniques applied to very-low-resolution thermal sensors did not distinguish between how background subtraction was being used to determine where a target was located versus how it was used to determine whether it was a valid target. This study evaluates these two scoring domains, background subtraction for location versus raw temperatures for evaluation, side-by-side on identical frames. In addition, operating continuously as a one-frame trigger allows the front-end to continue running indefinitely.

### 2.4. Triggered Sensing and Energy-Efficiency Metrics

The continuous power consumption of a fused stack is defined by its always-running members. Certified scanning and camera devices, together with their compute, draw watts continuously. The event monitored by such devices, a person entering the vehicle’s path, is rare. The gated architecture addresses this problem by dividing. There are two components to a divided system. The first component, a low-power sensor, monitors continuously. The second component, the high-cost stages, is only activated when required. This division is established practice in perception systems. In surveillance networks, distributed PIR front ends activate camera nodes [41]. Low-resolution thermal arrays have gated pose-detecting software running on an embedded platform. These systems achieve as much as 30% reduction in power usage in low-activity scenarios [80]. Edge machine learning uses hierarchical inference to replicate the division. Small always-on models serve each input. Large models run only on those inputs the smaller model cannot make decisions about [81]. Wake-up receivers are used with radio communications. One survey documented 132 commercially available component prototype implementations of this concept built from commercial off-the-shelf components [40].

None of the above branches provide a score for the collision avoidance designer that is required in order to choose a trigger. Wake-up detector performance is described through three measures: power consumption, detection accuracy, and false positive rate [39], all of which are provided as separate trigger characteristics. These values are trigger parameters and do not indicate the stack-level savings realized through gated deployment. Savings related to gated deployments were reported as a per-system percentage; however, the components included within those calculations are not defined [80]. Accuracy, latency, and energy, expressed as triples per platform, have been reported in hierarchical inference studies [81] but do not directly transfer to another stack. Analytical work addresses this problem from three perspectives. Each perspective uses analytical methods to optimize a design. None of them compare a measured system to an always-on baseline system. Stage threshold assignment based upon design-time expected computational expense represents one approach used in cascade models [82]. Another example includes scheduling policies that determine which sensors will be activated given an energy constraint [83]. In addition, analytical models of wake-up radios merge detection probability and false-alarm probability with an energy constraint for the communication rendezvous of a radio pair instead of a sensing operation [84]. Composite metrics score computation using the energy-delay product as the standard form [85]. They contain no measurement for task quality retained by a gated sensing stack. In addition, there are no defined scores for sensing triggers when they are tested only on their individual task, and the results are then related back to the power savings provided by the stack that the sensor gates.

### 2.5. The Gap

This exact pairing of sensors has been used before. They have been fused for near-field gesture interaction [86], proposed for use in person detection as part of a calibration study [87], and the same sensors have been used to detect people and other living beings at the camera tier since 2013 [68,88]. The open problems therefore lie in how such a trigger is evaluated and what it is worth. What is not known is the following: (i) the operational envelope of thermopiles when viewing horizontally, (ii) how to score detection without bias, and (iii) a figure of merit that relates trigger detection rates to the power saved by gating.

The first gap is the operational envelope in a horizontal view. Virtually all studies using low-resolution thermopile arrays have evaluated them viewing from above, resulting in relatively large targets and few confounding variables [58,59,62]. The closest to our work is a study of an 8 × 8 thermopile array in a horizontal view [63], but the maximum tested range is 3 m, and the array is only compared to an unheated source that produced no cluster. A full evaluation would include: (i) a thermopile array in the 8 × 8 class, (ii) viewing horizontally, (iii) viewing past 3 m, and (iv) a negative that is a passive thermal mass presenting a body-shaped warm cluster. Testing must take place in thermally distinct environments. There has not yet been a study of stationary human detection using all of these. The operational envelope also has a lower bound due to geometry. When the target width drops below one pixel, but the body height still fills the two-pixel minimum cluster, detection becomes scene-dependent. The field of view and number of pixels can be found in the datasheet and, together with the target dimensions and the detector’s minimum cluster size, used to develop a theoretical operational envelope. We are not aware of any work in the thermopile literature that develops an operational envelope based on sensor geometry or develops an envelope that can be applied to thermopile arrays with different specifications.

The second gap is scoring bias, which determines how believable a measured operational envelope is. In order to fairly assess the ability of the sensor to discriminate between classes, the data used in the discriminator must be independent of the data selected by the localizer. However, the background-subtracted residual is the basis of target localization as well as the commonly accepted scoring technique [76,77,78]. Selecting the best candidates with the localizer will bias the score towards improved class separation. None of the formulations we have seen restricts the background subtraction to localization and implements a score using the raw temperatures.

The third gap is a figure of merit for gated power. There is no metric in the existing literature which relates trigger detection rates measured empirically with the average power its fused stack saves. Related work on rendezvous in wake-up radio systems provides an analytical energy model that incorporates target detection and false-alarm probabilities [84]. Other works on cascades, hierarchical inference, and task scheduling have similar models for optimization at design time [81,82,83]. Other works that report energy savings with gated perception report the percentage of energy savings for their particular sensing systems as stand-alone results, without making it clear what parts of the system they consider [80]. In general, to develop a figure of merit for gated power, the savings have to be specified against a constant always-on reference, at a declared scope, and must carry a term for the task quality that gating preserves.

To this end, this paper makes the following contributions which address the gaps in sequence. It establishes the 8 × 8 horizontal operational envelope and computes its geometric lower bound with a coverage analysis. It presents an algorithm that scores on the original thermal data after localizing on the background-subtracted residual image. Finally, it develops a means to translate trigger-detection rates into power conserved at the system level.

## 3. Sensor Design

### 3.1. Hardware Design

The detection platform utilizes a single sensor device. It contains two sensors, one thermal and one distance, together with an inexpensive microcontroller. The Panasonic AMG8833 (Panasonic Industry Co., Ltd., Osaka, Japan), mounted on an Adafruit breakout board (Adafruit Industries, New York, NY, USA), provides thermal measurement. The STMicroelectronics VL53L1X (STMicroelectronics, Geneva, Switzerland), mounted on an Adafruit breakout board (Adafruit Industries, New York, NY, USA), provides time-of-flight distance measurements. The Arduino Nano (Arduino S.r.l., Monza, Italy) provides the processing capability. The Arduino Nano reads the data from both the Panasonic AMG8833 and the STMicroelectronics VL53L1X using a 400 kHz I^2^C bus. The data from the Panasonic AMG8833 are transmitted to the laptop over a Universal Serial Bus (USB) serially, in a single row of a CSV file per frame of data. All parts of this node draw less than 200 mW. The entire node would cost approximately USD 55 if purchased with commodity breakout boards. Our past research into distributed sensing and the system-of-systems architecture is what has led us to take such care regarding the cost and energy consumption for each node. Power consumption per node, and the unit cost, are the two major constraints that determine if a sensor network can be deployed at the scale its application requires [89,90,91]. A component’s measured benefit becomes comparable across different architectures only once its operational scope has been declared explicitly [92].

Of the three components, only the AMG8833 participates in thermal detection. This sensor is an 8 × 8 thermopile array with a 60° × 60° field of view. The sensor quantizes temperature at 0.25 °C and operates at a 10 Hz frame rate natively. The sensor has approximately 0.5 °C NETD [33], the minimum detectable temperature difference. The AMG8833 column of Table 1 lists all of the detailed specifications.

The AMG8833 cannot measure distance at all. The VL53L1X measures absolute distance. The VL53L1X is a single-zone ToF (time-of-flight) rangefinder using a 940 nm vertical-cavity surface-emitting laser (VCSEL) source and a default 27° field of view. With calibration, the VL53L1X provides measurement ranges from 40 mm to 4000 mm with ±3% accuracy. The remaining specifications are provided by the VL53L1X column of Table 1. At the system level, the VL53L1X will provide distance measurements to any downstream collision avoidance stack. In all trials, the ground-truth distance came from a tape measure.

**Figure 2:**
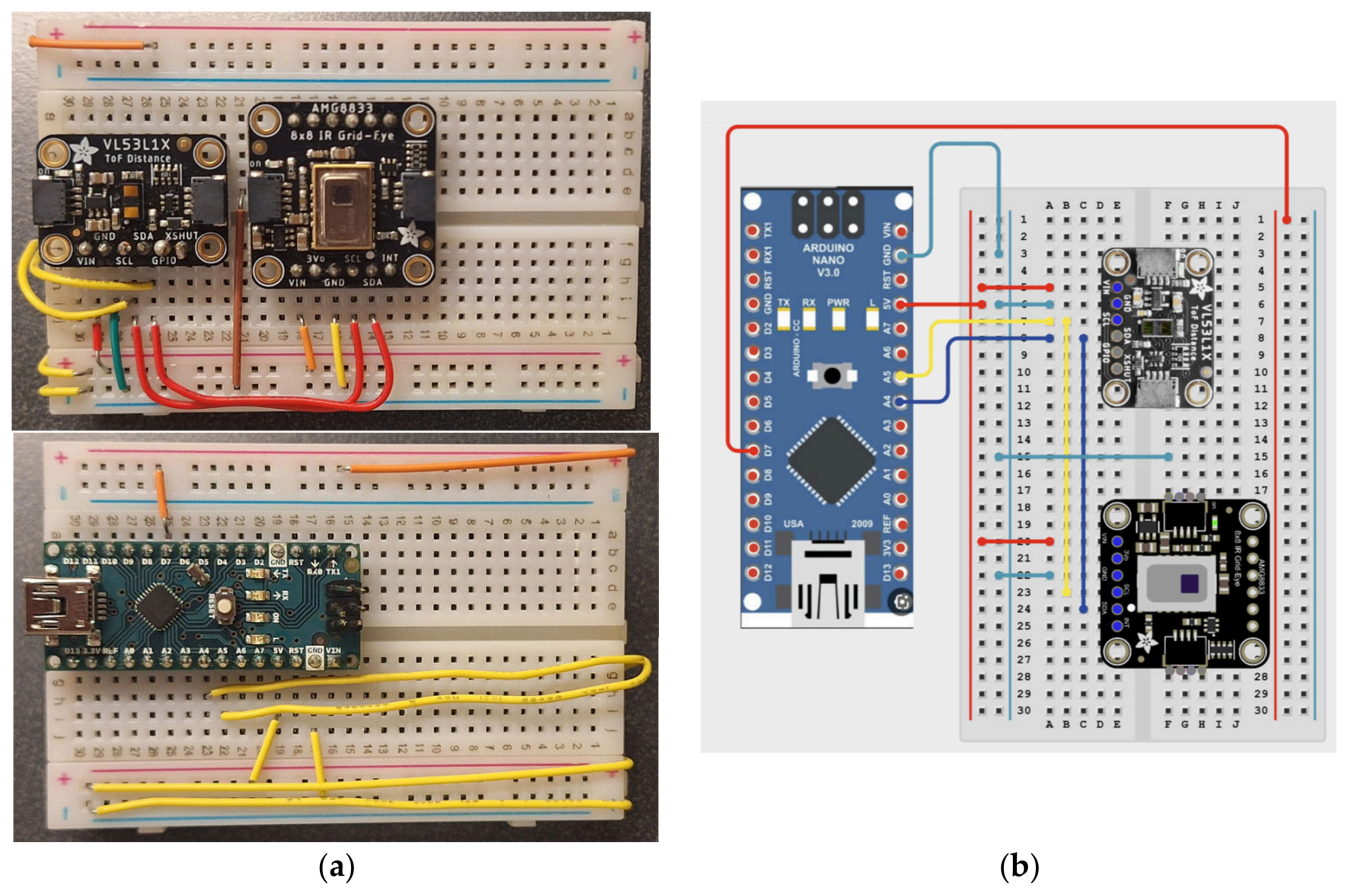
Firefly sensor node. (**a**) Assembled prototype front view and back view; VL53L1X time-of-flight rangefinder and AMG8833 thermopile array co-mounted with Arduino Nano on breadboard. (**b**) Wiring diagram. Both breakout boards share the I^2^C bus and are powered from the Arduino Nano’s 5 V USB rail.

In addition to how they are connected (bus topology), Figure 2 also illustrates the physical layout of the equipment listed in Table 2. The 5 V USB rail supplies power to the Arduino Nano. Both breakout boards are powered from the Arduino Nano’s 5 V rail and share its I^2^C bus. All three devices share a common ground. The sensor node is mounted on a tripod and held at a constant height. The optical axis of the AMG8833 is oriented horizontally. Therefore, the upper portion of its field of view looks above the subject towards the ceiling, sky, or distant background; consequently, the algorithm can use this as an ambient reference for the background in each thermal frame.

### 3.2. Target Angular Subtense and Pixel Coverage

The 60° angle along the two axes, vertical and horizontal, is sampled by 8 pixels on the AMG8833. The distance at which a target is spanned by exactly one pixel is calculated as (1):

(1) $$d_{1px}=\frac{w}{IFOV}$$

where $d_{1px}$ is that distance and w is the lateral width of the target. The instantaneous field of view per pixel (IFOV) is defined as 60°/8 = 7.5° = 0.1309 rad. This represents the smallest angular feature resolvable by the sensor. It is fixed by hardware and is independent of any algorithmic choice.

**Figure 3:**
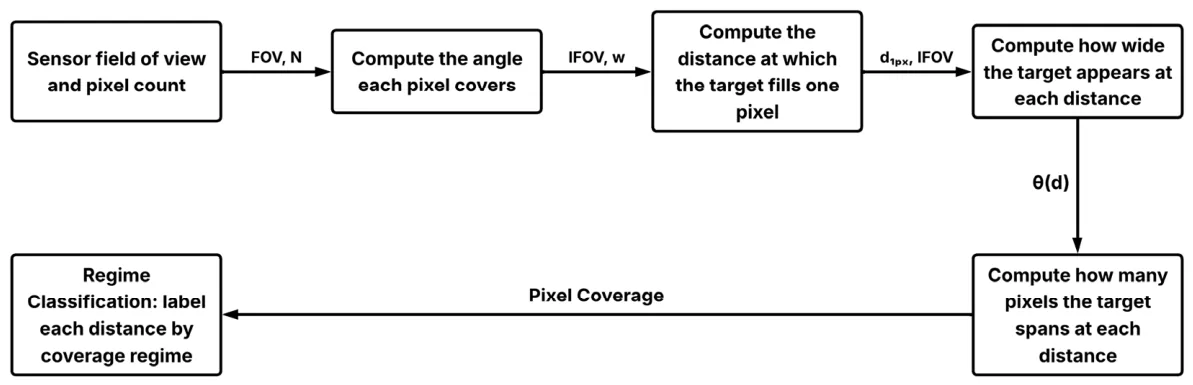
Pipeline mapping sensor field of view and pixel count through per-pixel IFOV, single-pixel fill distance $d_{1px}$, angular subtense θ(d) to pixel coverage, and coverage-regime classification at each test distance.

The target covers an area larger than a single pixel until $d_{1px}$, and at least one of those pixels is seeing nothing but the target. Beyond $d_{1px}$, the target will cover less than a full pixel in size. Since every pixel which does overlap with the target can see what is behind the target, no pixel will see just the target. This relationship creates three different zones for testing based on distance: multi-pixel ($d\;<\;d_{1px}$), fractional-pixel ($d\;\approx \;d_{1px}$), and sub-pixel ($d\;>\;d_{1px}$). Figure 3 shows how the data flow through each step of this process, from sensor specifications to coverage by distance to regime classification.

The vertical axis adds to this account. The detector needs at least two pixels next to each other, and a standing body is much taller than it is wide. For a subject of height h, the height subtends $\frac{h}{d\cdot IFOV}$ pixels at distance d. A cluster of at least two pixels therefore remains fully subtended out to $d_{2px}=\frac{h}{2\cdot IFOV}$, which is about 6.7 m for the 1.75 m test subject in this study. That is well beyond $d_{1px}$ = 4.2 m, the distance at which a 0.55 m body width falls below one pixel. Between $d_{1px}$ and $d_{2px}$, there is no single pixel which views the entire width of the target. Nonetheless, a two-pixel cluster could be formed. This cluster would consist of two pixels, vertically aligned, one directly above the other, with each being partially filled by the target. The vertically aligned pair will form a cluster if the reduced target-background contrast remains detectable. Beyond $d_{2px}$, even this vertically aligned pair is only partially filled. Therefore, the detection of targets will depend solely upon the contrast. Thus, geometry determines when detection will become dependent upon the scene as opposed to when detection ceases, which is the pattern the measured envelope shows.

### 3.3. Software and Algorithm Design

#### 3.3.1. Sampling Setup

The sensor node captured data from the AMG8833, which runs at its native 10 Hz frame rate. In every frame, the Arduino Nano read the thermal data in °C from all pixels of an 8 × 8 thermal matrix from the AMG8833. In addition to this, it polled the VL53L1X for a single value of time-of-flight distance in mm. Data were stored as csv files, with one row per thermal frame. The csv file included a time stamp for each thermal frame, the temperature reading of each of the 64 thermal pixels, and the measured time-of-flight distance for that frame. These csv files were saved for offline post-processing. Each trial consisted of 19 to 78 thermal frames. Each thermal frame was evaluated by itself to determine whether or not it contained a human.

#### 3.3.2. Per-Trial Frame Averaging

For each trial, we define a set S to represent all frames from the trial, where $S=\left\{F_{1},F_{2},\ldots ,F_{n}\;\right\}$. The averaged thermal matrix $\bar{F}\in R^{8\;\times \;8}$ can be calculated using (2):

(2) $$\bar{F}=\frac{1}{n}\sum_{k=1}^{n}F_{k}$$

where $F_{k}\in R^{8\;\times \;8}$ represents the k-th frame within S. Each pixel in the average thermal matrix $\bar{F}$ contains the mean for that pixel over all n frames in the raw temperature data.

#### 3.3.3. Per-Frame Analysis and Setup for F1 and SNR

Every thermal matrix $F_{k}$ has an orientation that follows the standard configuration. Row 0 will be oriented above the subject, towards the ceiling, the sky, or distant background. Before analysis, the human recordings and all factory recordings were rotated by 180° so that row 0 lies above the subject. The mannequin recordings from the lab, room, and outdoor trials were used as recorded. A thermal silhouette of the subject, when it is visible, lies in the middle-to-bottom area. The per-frame background ${BG}_{i}$ is defined as being the average value of the 8 pixels contained within row 0 of the raw frame $F_{k}$, as shown in (3):

(3) $${BG}_{i}=\frac{1}{8}\sum_{c=0}^{7}F[0,c]$$

where F[0, c] represents the temperature (in °C) of the pixel in row 0 at column c. Column index c varies from 0 to 7.

The amount that the background itself varies is measured by the same 8 pixels, and that is referred to as σ. σ is defined here (Equation (4)) as the spatial spread of those 8 values, floored at a minimum:

(4) $$\begin{array}{cccc} & \sigma =max(\sigma_{row},\;\sigma_{min}) & & \end{array}$$

where $\sigma_{row}$ is the spatial standard deviation of values for the 8 pixels in row 0. There is also a floor, $\sigma_{min}\;=\;0.05$ °C. This floor represents approximately one fifth of the quantization step. The reason this floor exists is so that σ will always remain positive regardless of whether $\sigma_{row}$ goes to zero because all eight pixels have an identical value.

#### 3.3.4. F1 and SNR

**Figure 4:**
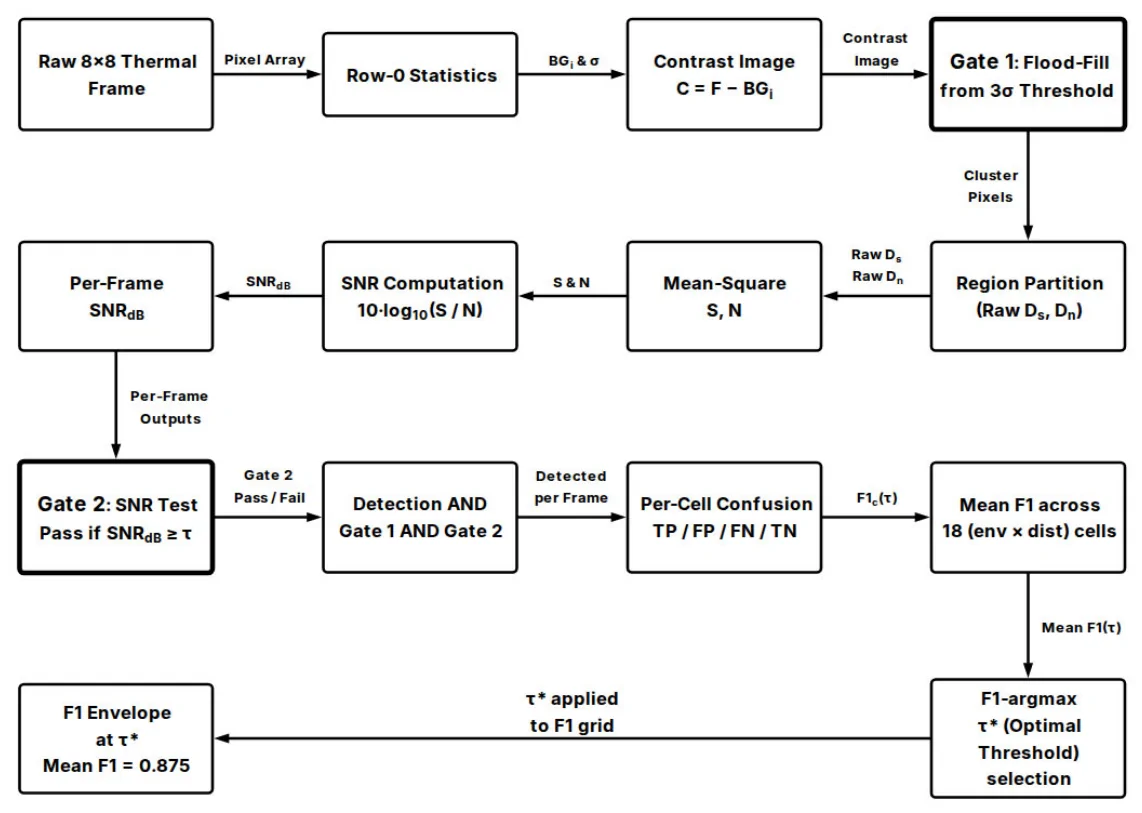
Two-gate detection pipeline: Gate 1 localizes a warm cluster via 4-connected flood fill on the background-subtracted contrast image, Gate 2 applies a threshold τ to the Celsius-scale SNR score, and the F1-argmax over all 18 environment–distance cells (lab, room, outdoor) selects the optimal operating point $\tau^{*}$.

The discrimination pipeline is a two-gate decomposition of the 8 × 8 frame, as shown in Figure 4. Gate 1 operates on the contrast image C as shown in (5):

(5) $$C\left[r,c\right]=F\left[r,c\right]-{BG}_{i}$$

The value of $C\left[r,c\right]$ is greater than zero when the pixel is warmer than the ambient temperature. The Gate 1 threshold is computed as in (6):

(6) $$\theta_{G1}=3\sigma$$

where σ is the per-frame noise estimate from (4). The “3” in the equation represents the standard 3σ outlier rule. A pixel is considered warm only if its temperature exceeds the background mean by more than three times the background’s own fluctuation. Gate 1 will start with the warmest pixel. This pixel is the position where the value of $C\left[r,c\right]$ is at the peak. If this peak value does not exceed $\theta_{G1}$, no new clusters will form, and Gate 1 will fail. On the other hand, Gate 1 will expand a new cluster from the initial warmest pixel by applying a 4-connected flood fill. Starting with the warmest pixel, Gate 1 will add all neighboring pixels that are orthogonally adjacent (i.e., above, below, to the left and/or to the right) whose contrast exceeds $\theta_{G1}$. Then, Gate 1 will check whether these new pixels qualify for inclusion in the cluster. Once again, they do so only if their contrast exceeds $\theta_{G1}$. The process continues until there are no longer any additional qualifying pixels. Gate 1 passes when the cluster reaches at least 2 pixels (K=|cluster|≥2), which rejects single-pixel noise. However, if Gate 1 fails, we reject the current frame. As such, the detection decision is 0. We will then set the SNR to 0 dB, and Gate 2 is not computed.

When Gate 1 passes, the 64 pixels are divided into two sub-regions. The cluster determines which sub-region will be defined as the signal region, $D_{s}$. The signal region includes all K pixels from the cluster. The noise region, $D_{n}$, includes all of the other 64  K pixels of the same 8 × 8 frame. Once these two regions are determined, Gate 2 computes the mean-squared temperature of each region, S for the signal region and N for the noise region, as given in (7) and (8):

(7) $$S=\frac{1}{K}\sum_{(r,c)\in D_{s}}F{\left[r,c\right]}^{2}$$

(8) $$N=\frac{1}{64-K}\sum_{(r,c)\in D_{n}}F{\left[r,c\right]}^{2}$$

S and N are computed in degrees Celsius, the scale the AMG8833 reports. Their ratio is an empirical decision score for Gate 2, referred to here as the SNR score. It is not a radiometric power ratio. Because the temperatures are squared before the ratio is taken, the score depends on the zero of the temperature scale. The SNR score in decibels is calculated in (9):

(9) $${SNR}_{dB}=10{log}_{10}\left(\frac{S}{N}\right)$$

A detection is made on a frame once Gate 1 has passed and ${SNR}_{dB}\;\geq \;\tau$. In this case, τ represents the threshold for Gate 2 and is defined on the Celsius-scale score. For a given value of τ, each frame’s decision is compared against ground truth. Ground truth is determined by what type of trial produced the frame: a human trial or a mannequin trial. All frames from the 36 trials are aggregated into a 2 × 2 confusion matrix. Each frame is counted individually. When an individual frame was generated during a human subject trial and is detected = 1, it is classified as a true positive (TP). When an individual frame was generated during a human subject trial and is detected = 0, it is classified as a false negative (FN). When an individual frame was generated during a mannequin trial and is detected = 1, it is classified as a false positive (FP). When an individual frame was generated during a mannequin trial and is detected = 0, it is classified as a true negative (TN). The standard binary classification metrics follow in (10):

(10) $$F1=\frac{2TP}{2TP+FP+FN}$$

The counts (TP, FP, and FN) are over all 36 trials at threshold τ. Threshold τ is swept from 0 to 10 dB in 0.1 dB increments. $\tau^{*}$, the single best value for maximizing F1 over all 18 environment–distance cells, is taken. $\tau^{*}$ is used for every environment and distance. Results are presented at that operating point. $\tau^{*}$ is chosen on the lab, room, and outdoor trials only. The factory environment trials are then scored with the same $\tau^{*}$, so they show how the detector performs on data that were not used to choose it. Trials using a human and a mannequin are compared across the 18 environment–distance cells. A two-sided sign test and a Wilcoxon signed-rank test are performed on the paired ΔSNR values. The unit of analysis is the cell and not the frame. The six distances within each environment are not independent of one another. Therefore, these tests establish the consistency of the direction rather than an effect size. Additionally, every frame is assessed using a second scoring method. In this second method, S and N are computed on the contrast image C rather than directly on the raw temperatures. That is the residual-scored configuration of [76,78]. The way in which Gate 1, the threshold sweep, and all reporting occurs does not change under that baseline.

### 3.4. Gated-Fusion Efficiency Formulation

Our system pairs two sensors: a low-power always-on thermal detector with active power $P_{t}$, used as a wake-up trigger, and a co-located time-of-flight (ToF) rangefinder with active power $P_{B}$, turned off until it receives a trigger. The host microcontroller runs always on. A gated sensor’s duty a∈[0,1] is the probability that its trigger fires. For our case, where the sensor is triggered by the presence or absence of a person, that is, binary detection, the duty can be determined using the true positive rate (TPR), the false positive rate (FPR), and the event occupancy λ∈[0,1]. The event occupancy is the fraction of frames in which a true event, a human, is present. The gated sensor’s duty is then computed as (11):

(11) a(λ) = λ TPR + (1 − λ) FPR = FPR + (TPR − FPR) λ.

#### 3.4.1. Average-Power Model for Triggered Operation

The account generalizes from one gated sensor to a triggered multi-sensor system with M sensors. The gating policy activates a subset A of these on each input. Both the power drawn and the quality delivered are then expectations over A. The average power is the expected power consumption of the set of sensors that have been activated, as in (12):

(12) $$P_{avg}=E\left[\sum_{i\in A}P_{i}\right]\;=\;\sum_{i=1}^{M}a_{i}P_{i}\;+\;\sum_{j}r_{j}e_{j}$$

The always-on reference is a similar expression, as in (13), for all sensors in use:

(13) $$P_{full}=\sum_{i}P_{i}+\sum_{j}r_{j}e_{j}$$

where $a_{i}$ and $P_{i}$ represent the duty and active power of sensor i, respectively. The terms $r_{j}$ and $e_{j}$ represent the invocation rate and per-invocation energy associated with processing stage j. The task quality is the expected quality of decisions that will be produced by the active subset calculated in (14):

(14) $$Q_{gated}=E\left[Q\left(A\right)\right]$$

where Q(A)∈[0,1] represents the level of task quality that is achieved when the subset A is activated. It can either represent the baseline quality $Q_{base}$ that is present with only the root sensor active, or the full quality $Q_{full}$ that is achieved with all sensors activated.

#### 3.4.2. Energy Reduction, Quality Retention, and the Figure of Merit

Gated-Fusion Efficiency (GFE) measures how efficiently triggering converts power into task quality, against the always-on reference. This is accomplished through evaluating two different aspects. The first aspect is the energy-reduction factor calculated by (15):

(15) $$\Phi \;=\;\frac{P_{full}}{P_{avg}}$$

The energy-reduction factor is a representation of the number of times gated fusion uses less than the always-on average power. The second aspect is the quality retention calculated by (16):

(16) $$\tilde{\rho }\;=\;max\left(0,\;min\left(1,\;\frac{Q_{gated}\;-\;Q_{base}}{Q_{full}\;-\;Q_{base}}\right)\right)$$

Quality retention is a representation of the ratio of the improvement in quality from baseline to always-on that is retained when using gated fusion. Quality retention is equal to 0 at the baseline, and 1 at the always-on reference. Gated-Fusion Efficiency is the product of these two as in (17):

(17) $$\eta \;=\;\tilde{\rho }\cdot \Phi$$

where the product represents the savings in power Φ, times the quality retained $\tilde{\rho }$. Always-on operation is used as the base case at η=1, since it saves no power and loses no quality. Any time η>1 represents a net gain. A higher frequency of events causes the gated sensor to wake up more often, and therefore η will decrease. The occupancy at which η=1 marks the threshold past which triggering does not provide a benefit.

## 4. Experiments

### 4.1. Experimental Design

Experiments assessed how well the Firefly configuration could distinguish humans from objects with shapes that were very similar. The experiments crossed two subjects, six distances, and four environments, with three postures and three repeats added in the factory environment. Both subjects were the same for all experiments. A mannequin served as the negative class, while an adult male, one of the authors, served as the positive class. In the lab, room, and outdoor trials, he wore a T-shirt. In the factory, both he and the mannequin wore a hard hat and a high-visibility vest, as is typical on a factory floor. The human subject participated willingly. No personally identifiable data were recorded since the 8 × 8 array records only 64 temperatures per frame. The reason that a mannequin was chosen as the negative class is because it simulates a person’s silhouette.

**Figure 5:**
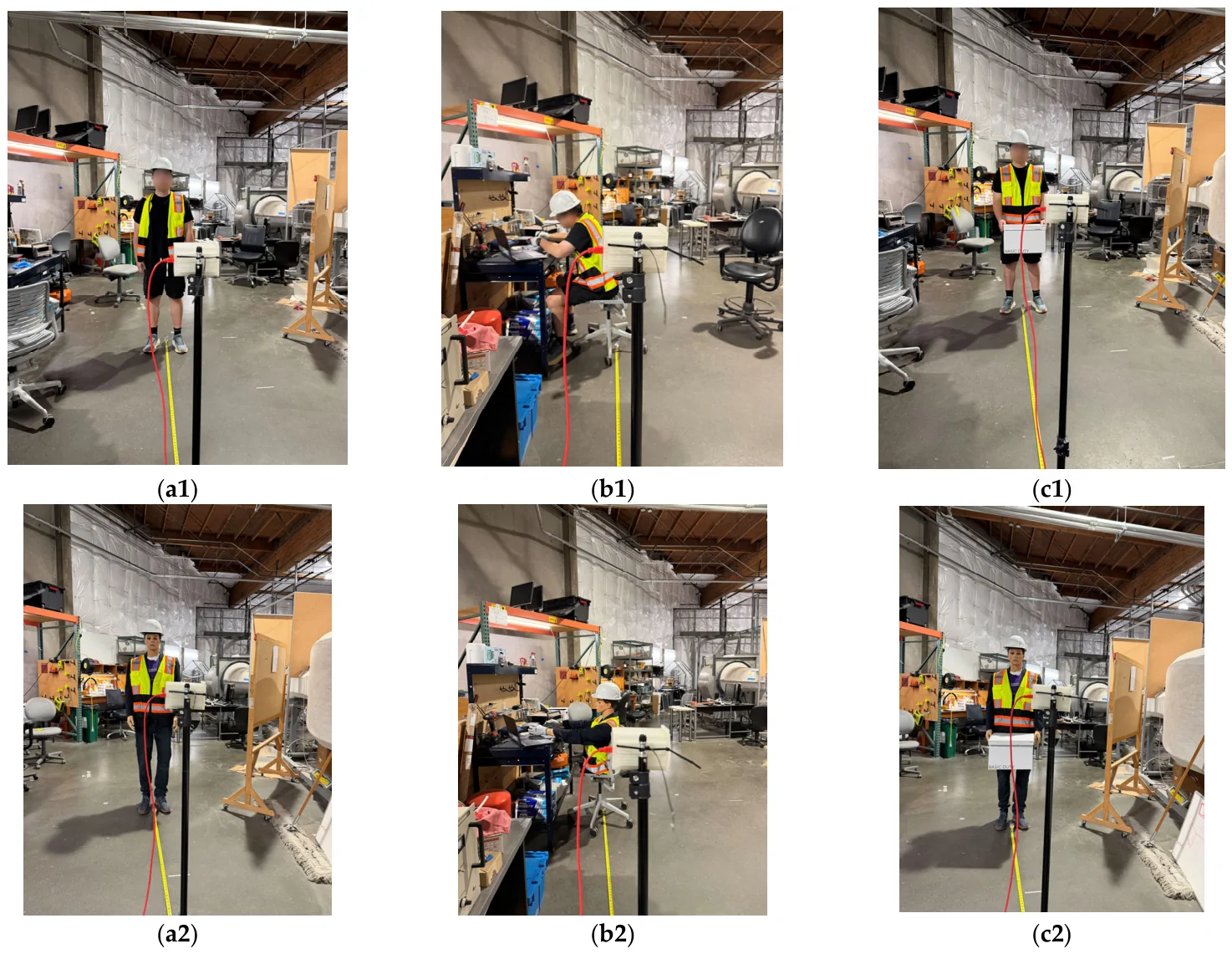
Factory environment experimental setup. (**a1**,**a2**): Pair of standing human subject and standing mannequin. (**b1**,**b2**): Pair of seated human subject and seated mannequin. (**c1**,**c2**): Pair of human subject and mannequin holding a box in front of the torso.

In all experiments, the subject was directly in front of the sensor. In the factory environment, two further postures were recorded in addition to standing: standing while holding a box in front of the torso and seated, as shown in Figure 5. The mannequin was placed in the same three postures, and every class, posture, and distance was recorded three times. The other three environments have one standing trial per class and distance. The sensor unit rested atop a tripod that was placed at approximately 1.4 m high off the ground. The test was carried out with six different measuring distances: 1.2 m, 2.2 m, 3.2 m, 4.2 m, 5 m, and 7 m. The span covers the near-vehicle scale at which a driverless industrial truck must stop and the larger scale at which it reduces speed on detecting a person ahead [27,93]. The distance was determined using a tape measure from the center of the tripod to either the toe of the subject, or the base of the mannequin.

Four different thermal environments were used for conducting the experiment. A laboratory setting provided a uniform and well-controlled background. A room environment added structured indoor clutter to the background, such as walls, corridors, and windows. Outdoor trials encountered mixed lighting, shadows, and large differences in spatial temperature throughout the scene. The fourth environment was the working floor of an operating factory. Together, these environments span a range of background conditions representative of realistic industrial and warehouse settings. Table 3 gives the range of pixel temperatures recorded in each environment.

### 4.2. Data Collection and Frame Counts

Thermal image data were acquired via a USB serial interface from the AMG8833 sensor, which runs at 10 Hz. For every trial, a comma-separated values (CSV) file is created as an output record. The csv file contains the elapsed time since the start of the trial in milliseconds, the distance from the subject to the thermal sensor in mm, and the temperature of each of the 64 pixels in the 8 × 8 thermal matrix. Every trial contains 19 to 78 frames. The total number of frames was 1379, across the 36 lab, room, and outdoor trials. A complete list of the number of frames can be seen in Table 4 (the 663 frames for a real human) and Table 5 (the 716 frames for mannequins). These two tables also list the factory trials, in separate rows for each posture. Each factory trial lasted 36 frames, so the 108 factory trials (two classes, three postures, six distances, three repeats) add 3888 frames.

## 5. Results

### 5.1. SNR Separation Between Human and Mannequin

**Figure 6:**
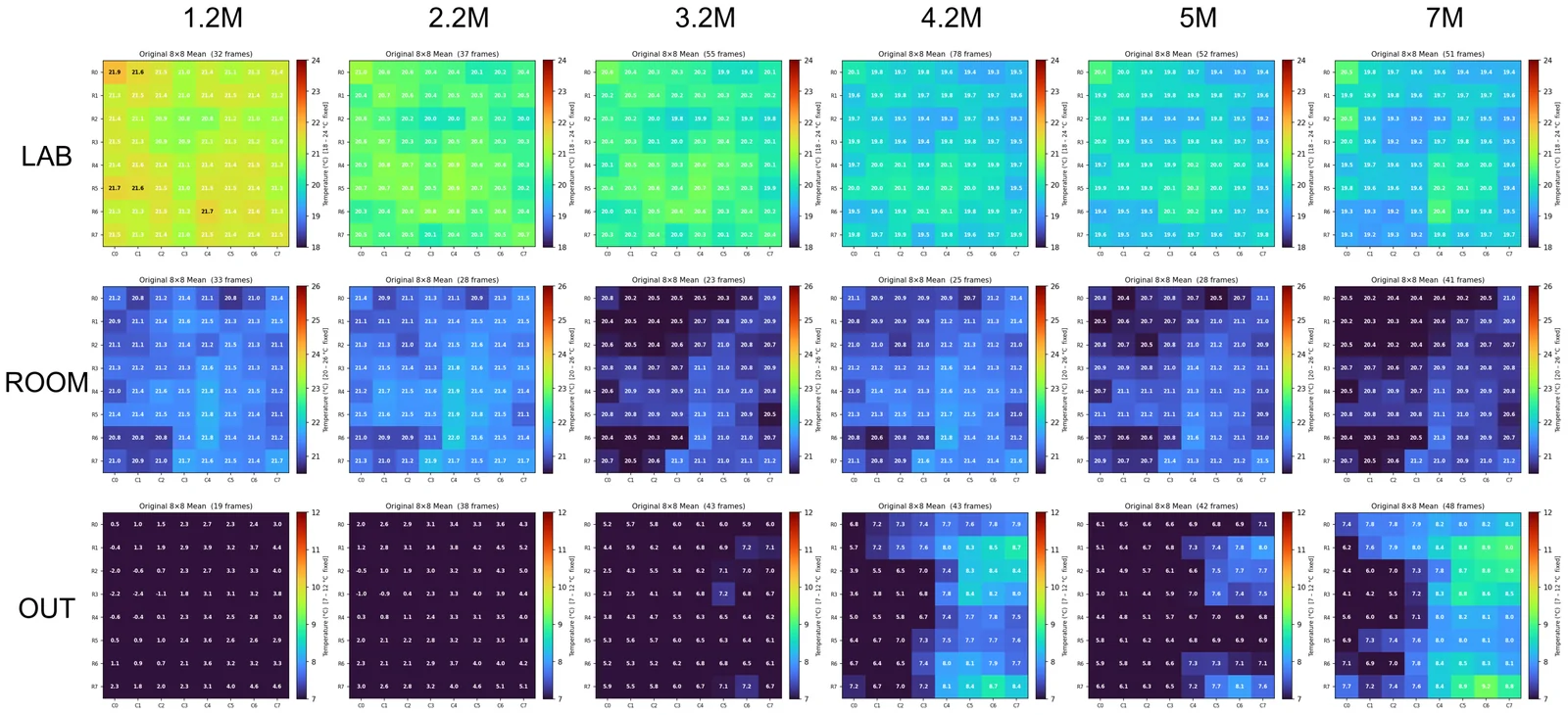
Mean 8 × 8 thermal frames (averaged across all frames per trial) from the AMG8833 for mannequin trials (negative class). Three environments (rows) × six distances (columns: 1.2–7 m). Color scale: mean pixel temperature (°C).

**Figure 7:**
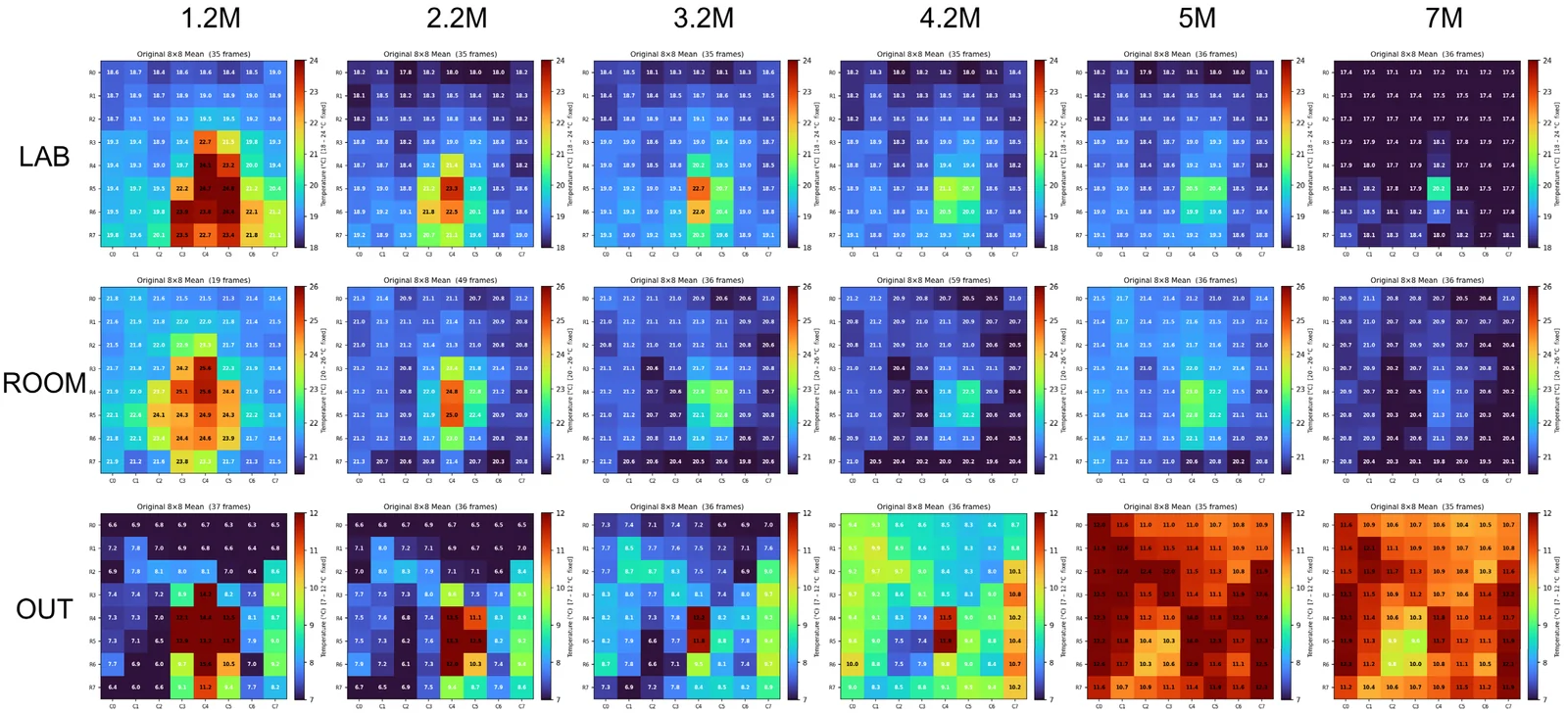
Mean 8 × 8 thermal frames (averaged across all frames per trial) from the AMG8833 for human-subject trials (positive class). Three environments (rows) × six distances (columns: 1.2–7 m). Color scale: mean pixel temperature (°C).

The data shown in Figure 6 and Figure 7 are the averaged thermal images of the mannequin and human trials. These are the thermal images of mannequins and humans at the three different environments and at the six different distances. Thermal signatures were greater in magnitude and more localized in the case of humans versus mannequins at most distances. The signatures of both mannequins and humans contracted toward a single pixel by 7 m.

**Figure 8:**
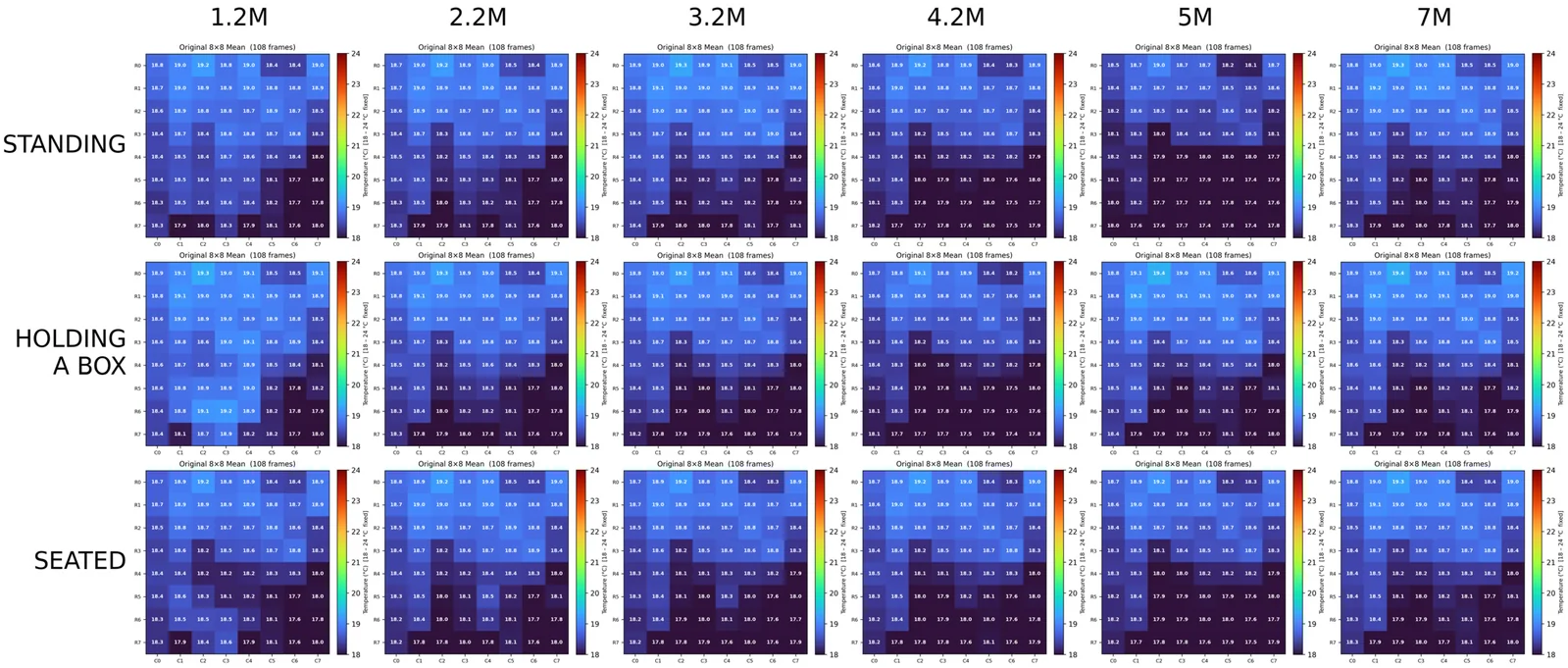
Mean 8 × 8 thermal frames (averaged across the 108 frames of the three repeated trials) from the AMG8833 for mannequin trials (negative class) at the factory environment. Three postures (rows) × six distances (columns: 1.2–7 m). Color scale: mean pixel temperature (°C).

**Figure 9:**
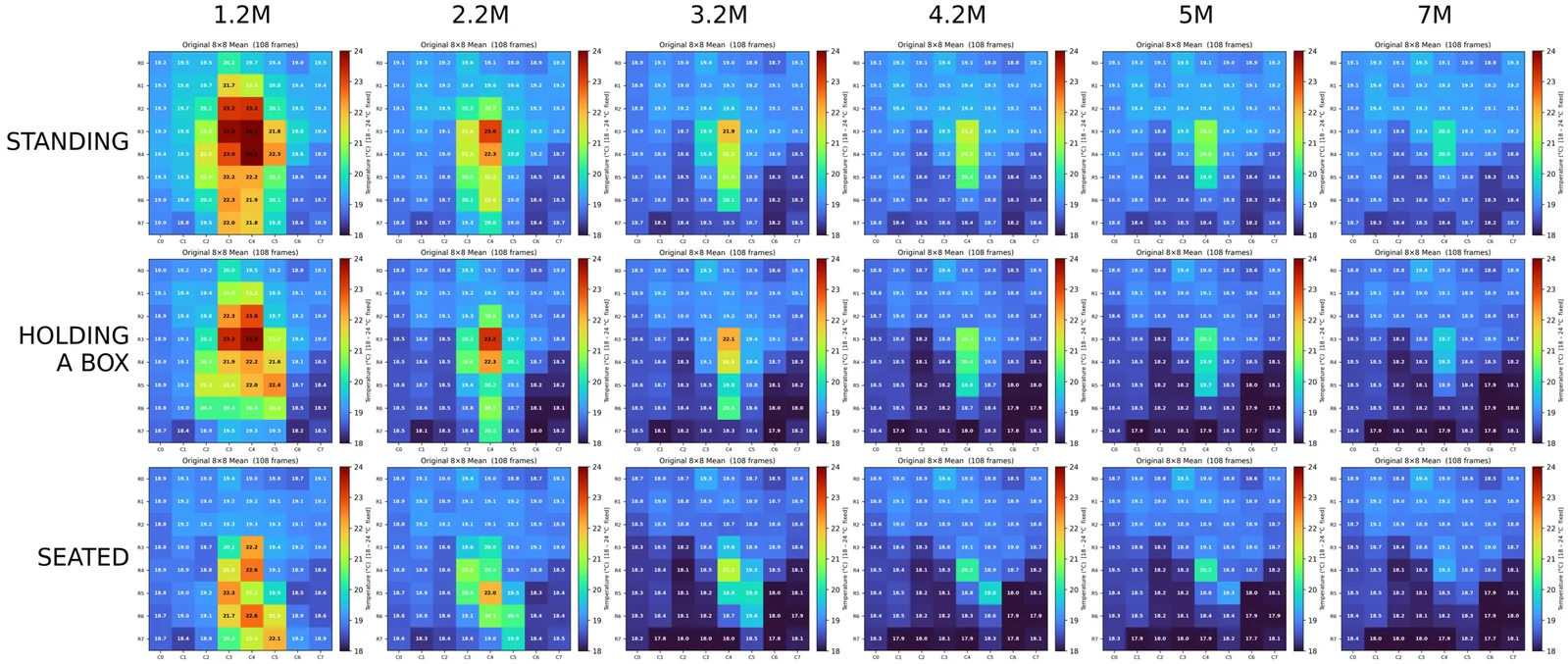
Mean 8 × 8 thermal frames (averaged across the 108 frames of the three repeated trials) from the AMG8833 for human-subject trials (positive class) at the factory environment. Three postures (rows) × six distances (columns: 1.2–7 m). Color scale: mean pixel temperature (°C).

The data shown in Figure 8 and Figure 9 are the averaged thermal images of the mannequin and human trials at the industrial site. These are the thermal images of mannequins and humans in the three postures and at the six different distances, averaged over the three repeated trials. The mannequin showed no warm region in any posture, while the human signature depended on the posture: it was largest when standing, was interrupted by a cooler row where the box covered the body, and was smaller and lower in the frame when seated. The human signatures contracted toward a single pixel by 7 m when standing or holding a box, and already by 4.2 m when seated.

The comparison between human trials and mannequin trials for each of the three environments and six distances was made by using the ΔSNR. The ΔSNR is defined as the difference between the mean ${SNR}_{dB}$ of the human trial frames and the mean ${SNR}_{dB}$ of the mannequin trial frames. All 18 values are given in Table 6. The ΔSNR is positive for 17 out of the 18 values, against the nine that would be expected if humans and mannequins could not be distinguished. Using a two-sided sign test, we find $p=1.4\times 10^{-4}$. Using a Wilcoxon signed-rank test on the magnitudes of the differences, we find $p=1.5\times 10^{-5}$. In the laboratory setting, the ΔSNR values ranged from +0.514 dB to +1.255 dB across all six distances. In the room setting, they ranged from +0.354 dB to +0.878 dB. The one exception was room 7 m, where the ΔSNR was −0.074 dB. In the outdoor setting, all ΔSNR values were positive, ranging from +0.261 dB at 7 m to +2.135 dB at 4.2 m.

### 5.2. Detection Performance

**Figure 10:**
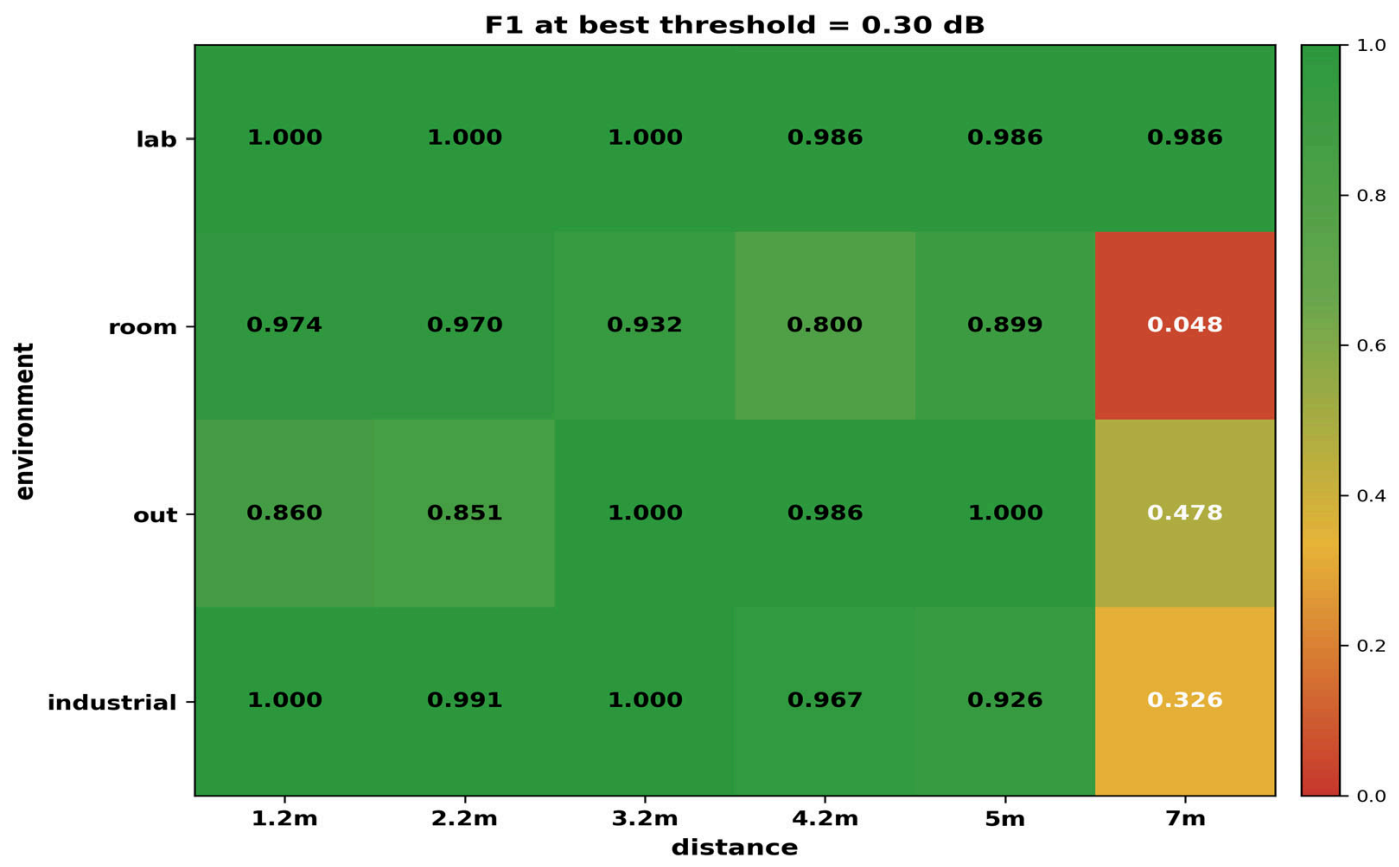
F1 from the standing human and mannequin trials at each environment and distance, at the operating point $\tau^{*}$ = 0.30 dB.

Table 7 and Figure 10 give the F1-values for each distance and environment. Each value was computed by combining results for the mannequin and human trials, with the threshold held constant at 0.30 dB. In the lab, there were no errors for the first three distances (F1 = 1.000 for 1.2, 2.2 and 3.2 m), values were ≥0.986 everywhere else, and the mean F1 was 0.993. In the room, F1 was ≥0.932 for distances of 3.2 m and less, ≥0.80 up to 5 m, and dropped off sharply to 0.048 at 7 m. The mean F1 was 0.770. Outdoors, F1 was 0.860 and 0.851 for 1.2 and 2.2 m, ranged from 0.986 to 1.000 between 3.2 and 5 m, and dropped off at 7 m (F1 = 0.478). The mean F1 was 0.863. An unweighted average over the 18 lab, room, and outdoor cells yielded an overall F1 of 0.875. In the factory, the subject was also standing, as in the other three environments, and the threshold was the same. F1 remained ≥0.926 up to 5 m and then decreased to 0.326 at a distance of 7 m. The average F1 was 0.868. The pooled confusion matrix calculated from all of the frames (N = 1379) in the lab, room, and outdoor environments contained TP = 577, FP = 43, FN = 86, and TN = 673, yielding precision and recall of 0.931 and 0.870. The overall F1 was 0.900 over the entire data set, excluding the factory trials. Note that consecutive frames are near-replicates as the subject was not moving, but that the number of frames per trial varied widely (see Table 4 and Table 5 for the number of frames in each trial, which ranged from 19 to 78). Because of this, the per-cell values, rather than the pooled ones, are the central findings of this research.

Table 8 lists F1 in the factory environment for the three postures. Standing, F1 stayed at or above 0.926 through 5 m. When the subject held a box in front of the torso, F1 stayed at or above 0.941 through 4.2 m and dropped to 0.633 at 5 m and 0.088 at 7 m. Seated, F1 stayed at or above 0.976 through 2.2 m, fell to 0.761 at 3.2 m, and was near zero from 4.2 m on. Both shorter ranges follow from Gate 1, which needs at least two adjacent warm pixels. A seated person presents a smaller warm region, lower in the frame, so from 4.2 m on, fewer than two adjacent pixels are warm enough. A box held in front of the torso covers the middle of the warm region and splits it into two parts, the head and shoulders above the box and the legs below it. From 5 m on, each part covers about one pixel, and neither forms a cluster on its own. The three repeated trials gave closely matching results. The mean F1 over the six distances ranged from 0.847 to 0.895 standing, from 0.761 to 0.783 holding a box, and from 0.442 to 0.472 seated. The repeats differed mainly at the distance where detection was already falling off. The mannequin at this site was at room temperature and triggered a detection in only five of its 1944 frames, fewer than in the other environments, so these results mainly reflect how well the human was detected.

**Figure 11:**
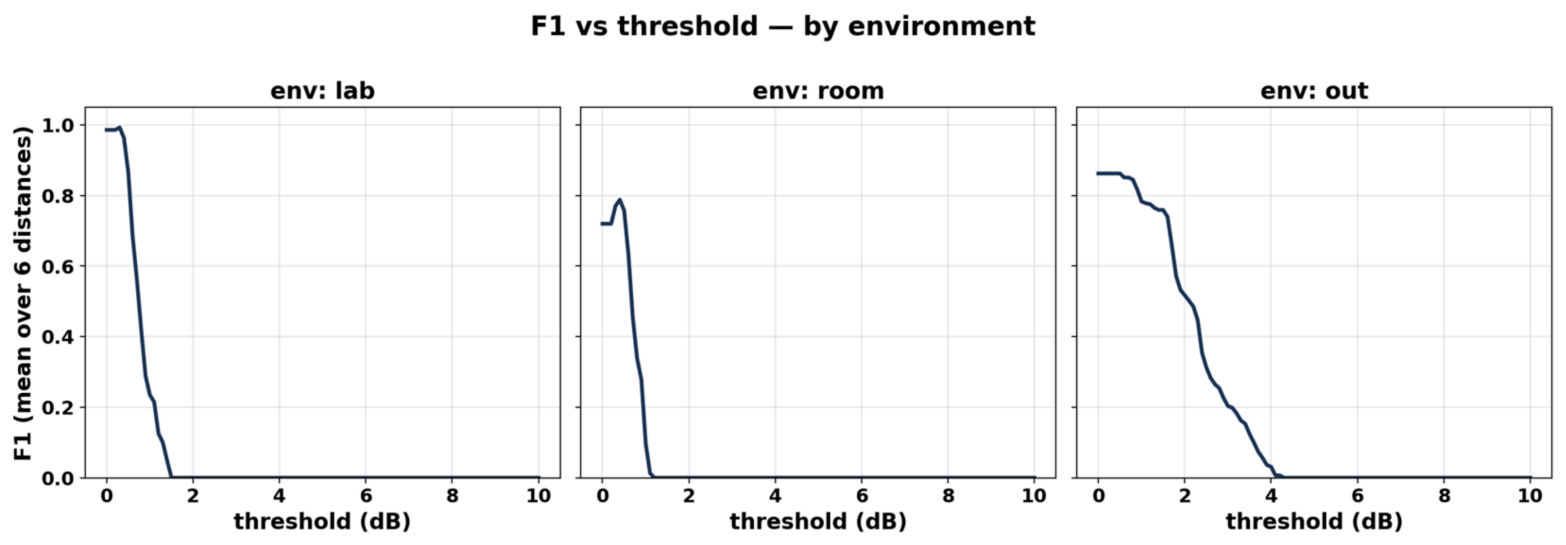
Mean F1 as a function of SNR threshold τ (0–10 dB), faceted by environment ((**left**) lab; (**center**) room; (**right**) outdoor). Each panel shows the unweighted mean of per-cell F1 across six test distances. *X*-axis: τ (dB). *Y*-axis: mean F1.

Figure 11 shows the F1-score as a function of thresholds, which range from 0 to 10 dB, for each of the environments (separated panels). More precisely, the curve for the lab environment, which has the sharpest decrease, is very close to 1 from 0 to 0.3 dB and decreases to 0.87 for 0.5 dB and 0 for about 1.5 dB. For the room, there is also a plateau followed by a sharp decrease, but at a lower level compared to that for the lab, namely, 0.72 from 0 to 0.2 dB, a maximum of 0.79 for about 0.4 dB, and a decrease to almost zero. Finally, for outdoors, the decrease is quite slow, namely, the curve starts with a value of approximately 0.86 for 0 dB, has a value of 0.76 for approximately 1.5 dB, and reaches 0 for about 4.3 dB. Although the shape of the three curves is quite different, an operating point of 0.30 dB is within the region of high F1-score for all of them.

In each case, performance is shown using an individually optimal threshold for F1, on a 0.1 dB grid. On that grid, the best mean F1 is 0.872 using residual scoring, or 0.875 with raw-temperature scoring. Note that the dB figures and threshold ranges in these results are not directly comparable, because the baselines have different zeros, with 0 °C for raw scores and the background for residual scores. Stability is therefore judged by the decrease in F1: using leave-one-cell-out thresholding, the decrease in mean F1 is 0.023 for the residual scores and 0.008 using the raw-temperature scores.

We also repeated this evaluation using the SNR score in kelvin, since the SNR score depends on the zero of the temperature scale. The scores in kelvin have a range of less than 0.15 dB, which is too small to use a 0.1 dB grid. In both units, the threshold was therefore selected by an exhaustive search, which places a candidate threshold midway between every pair of adjacent score values and keeps the one with the highest mean F1 over the 18 cells. This search places the threshold at 0.353 dB in Celsius and 0.022 dB in kelvin. These two thresholds serve only this comparison, and the operating point of 0.30 dB is unchanged. Only nine decisions for the 1379 frames were different using kelvin rather than Celsius, and in each case, the decision in Celsius was correct. The mean F1 was 0.882 in Celsius and 0.875 in kelvin. For the leave-one-cell-out threshold, the mean F1 was 0.881 in Celsius and 0.871 in kelvin. In kelvin, the ΔSNR was positive in 17 of the 18 cells of Table 6, and the sign test and Wilcoxon p-values are the same. Thus, based on these data, none of the conclusions would be affected by using the kelvin score. However, it is unreliable near 0 °C. In particular, for the outdoor mannequin trial at 1.2 m, the human and mannequin Gate-1-passing SNR frames are ordered the other way in Celsius compared to kelvin since the background was close to 0 °C. If the scene might be near or below freezing, the kelvin score is required.

### 5.3. Gated-Fusion Efficiency

We determine the gated-fusion efficiency using the measured thermal trigger gating the co-located VL53L1X. The only measured input to this process is the operating point of the trigger. With a 0.30 dB threshold, our pooled confusion matrix shows a true-wake rate of 0.870 and a false-wake rate of 0.060. These rates tell us how frequently the ToF will be active based upon occupancy levels. With a sampling frequency of 10 Hz, a false-wake rate of 0.060 translates into the ToF being active 6.0% of the time when there is no person in view, about 3.6 min per hour. The 0.060 value is the false-positive rate on mannequin frames, used here as an upper bound on the empty-scene rate.

**Figure 12:**
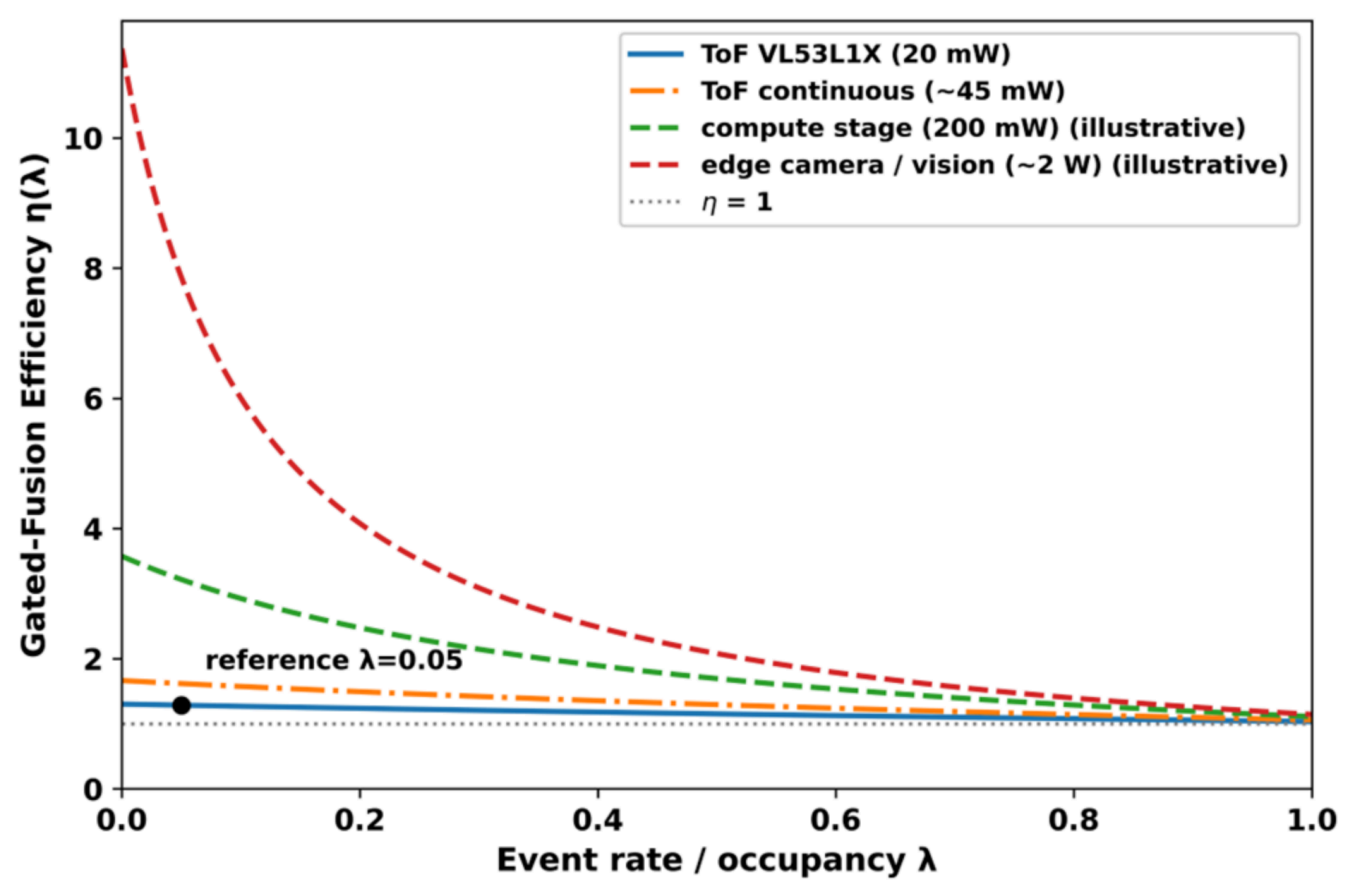
Gated-fusion efficiency η(λ), equal here to the energy-reduction factor Φ(λ), swept across the full occupancy range for four gated stages spanning two orders of magnitude in power ratio $\kappa =\frac{P_{B}}{P_{0}}$.

Occupancy is a parameter that indicates how often the event occurs. This is the number of frames with targets divided by the number of all frames. Occupancy is 0 for an empty scene and 1 for a scene that contains a person in every frame. Here, results are given for occupancy of 0.05, that is, a person in view for 3 min in every hour. This value was chosen because wake-up detectors are designed for such sparse events [39,41]. For validity, we still ran a sensitivity test on λ. Figure 12 shows each stage over the full range of occupancies. The thermal trigger is always on while the ToF sensor only uses energy when triggered, so its duty a(λ) depends on the occupancy λ. The duty grows linearly from a minimum, or false-wake rate, of 0.060 at λ=0 to the true-wake rate of 0.870 at full occupancy, λ=1, that is, a(λ)=0.060+0.810λ. This duty then enters the energy-reduction factor Φ(λ).

The ToF ranges only targets that the thermal trigger has already found. It wakes precisely on the frames that hold those targets. Frames where the trigger stays quiet need no ranging at all. Gating therefore gives up no task quality. The gated quality equals the always-on quality, so retention is one ($\tilde{\rho }=1$), and the efficiency reduces to the power saving alone.

The element powers represent data sheet values at defined operational conditions instead of in-circuit measurements. The always-on AMG8833 trigger consumes 15 mW [33], while the ATmega328P that runs the detection consumes 46 mW, that is 9.2 mA at Vcc = 5 V with a 16 MHz clock, which instantiates the $\sum_{j}r_{j}e_{j}$ term [94]. The gated VL53L1X consumes 20 mW at the specified 10 Hz/33 ms time budget, and 45 mW, that is 16 mA at 2.8 V, in continuous ranging [65]. Figure 12 shows how much efficiency can be expected from four different gated stages. They vary according to their power ratio κ, given by (18):

(18) $$\kappa =\frac{P_{B}}{P_{t}+\sum_{j}r_{j}e_{j}}$$

The power ratio indicates how much power is consumed by a sensor when it is gated, as opposed to the power consumed by the trigger and host, which are always on. Therefore, if the power ratio is small, the power draw is very similar to the trigger. If the power ratio is large, the power draw for that sensor is much larger. This indicates that we get a bigger power saving when we gate sensors which use more power. Indeed, the maximum possible stage efficiency η is $\frac{1}{a\left(\lambda \right)}$. At 5% occupancy, this is 9.9×, and stages which consume more power come closer to this. This makes sense since these stages will be powered up only the duty a(λ) of the time. None of them achieve this because of power consumption in the trigger and host. Therefore, the ToF sensor with a power ratio κ of 0.33 gains the least and sits near the bottom of the range, whereas the vision stage with a ratio of 32.8 sees the best gains close to the theoretical maximum.

Table 9 shows our results at 5% occupancy. At a budget of 33 ms, or 10 Hz, our measured ToF has an efficiency of 1.29× at a power ratio of 0.33. Removing the always-on 46 mW host moves the scope from node to sensor and leads to a power ratio of 1.33, hence, an efficiency of about 2.06. In contrast, ranging continuously achieves an efficiency factor of 1.62× at a power ratio of 0.74. Additionally, we estimate the power-saving factors for high-power accelerators that we do not have in our testbed. An edge neural processing unit (NPU) such as the Kendryte K210 [95], which is below 1 W and consumes approximately 200 mW, has a power ratio of 3.28 and hence savings of 3.22×. Similarly, a vision accelerator that consumes approximately 2 W, such as the Google Coral Edge TPU (tensor processing unit) [96], achieves a power ratio of 32.8 and a savings factor of 7.86×.

## 6. Discussion

### 6.1. Design Boundaries of Gated Fusion

The savings realized from gating are determined by two values. We group the always-on root $P_{0}=P_{t}+\sum_{j}r_{j}e_{j}$ and factor it out of the energy-reduction factor to get (19):

(19) $$\Phi \left(\lambda \right)=\frac{1+\kappa \;}{1+\kappa \;\cdot a(\lambda )},\;\;\kappa \equiv \frac{P_{B}}{P_{0}}$$

In general, the amount of power savings depends only on the power of the gated sensing stage relative to that of the root, κ, and its duty-cycling behavior. Since the root consumes power on every frame, the maximum possible saving is bounded by 1 + κ. The hardware ratio alone therefore caps the reduction.

The trigger sets a second cap. The duty a(λ)=FPR+(TPR−FPR)·λ cannot fall below the false-positive rate FPR, so at every occupancy satisfies (20):

(20) $$\Phi \left(\lambda \right)\leq \Phi_{max}=\frac{1+\kappa \;}{1+\kappa \;\cdot FPR}$$

There are two limits to the savings from gating, and the product κ·FPR determines which one binds. One limit is set by the hardware ratio. The other is set by the false-positive rate of the trigger. In cases where the product is much less than one (κ·FPR≪1), the hardware limit applies, and the maximum possible saving is approximately $\Phi_{max}\approx 1+\kappa$. At those values, false positives cost essentially nothing. Conversely, in cases where the product is either near or greater than one (κ·FPR≳1), the trigger limit applies, and the maximum possible saving $\Phi_{max}$ approaches $\frac{1}{FPR}$. No gated stage operating at any level of power behind a single trigger can have its reduction exceed $\frac{1}{FPR}$. As measured for the trigger tested here, 1/FPR is a factor of 16.7×.

Next, consider how much saving is possible given our example trigger characteristics. The always-on root uses 61 mW at the node. The measured ToF, the gated stage here, has a power ratio κ of 0.33 relative to that always-on root. The product κ·FPR is 0.02, low enough that we are hardware-capped. A cleaner trigger would buy almost nothing. Indeed, the saving is 1.29× for an occupancy of 0.05, against the hardware limit of 1 + κ = 1.33×. Doubling the false-positive rate decreases the savings from 1.29 to 1.26. Halving the true-positive rate still gets 1.29. As an extreme example of what happens when the trigger is the limit instead of the hardware, suppose a much higher power vision stage with a budget of 2 W. That would be a power ratio κ of 32.8 relative to the always-on root of 61 mW, with κ·FPR of 2.0. Now, we are trigger-capped and get maximum possible savings of 11.4× for zero occupancy, even though the hardware ratio (1 + κ) suggests savings of up to 33.8× could be possible.

The caps translate into selection rules for all multi-sensor systems where a stage is gated. To gate a given stage with a trigger, the trigger’s false-positive rate must be much smaller than the inverse of the power ratio. In the trigger-capped regime, the best possible reduction scales inversely with the false-positive rate. Improvements in the false-positive rate therefore pay off linearly. In this regime, improvements to the gated stage such as making it cheaper result in no improvement at all. When no single trigger reaches a low enough false-positive rate, multiple stages can be cascaded: a second gated stage is activated only when the first fires. The duty is the product of the duties at each stage, so the last stage is presented with a tiny fraction of the original set of sensor frames. In this way, a set of modest triggers can gate a stage that no single trigger could.

The quality-retention term $\tilde{\rho }$ categorizes triggered architectures into two types, each type having a different gating verdict. For architectures of the first type, the gated stage only refines what the trigger has previously identified, as ranging does for detection here, and so it retains all task quality when gating occurs. Retention is therefore $\tilde{\rho }$ = 1 and the efficiency is at least η=Φ≥1. The gated stage benefits at all occupancy levels, so it should be gated unconditionally. Quality retention falls below one ($\tilde{\rho }<1$) if the gated stage resolves a portion of the input space that was missed by the trigger. In this case, there is a break-even point with respect to occupancy, above which always-on fusion wins. Gating therefore pays off only in those scenarios where the scene is sparse. In both of the described categories, whether or not a stage should be gated is based solely upon its own quality retention and duty level, regardless of how much always-on power is available on the processing platform. This independence provides for portability of gating decisions across platforms. The reported efficiency η represents the overall impact of gating at the scope that was declared. Declaring the scope is what allows comparison of efficiencies among different systems.

### 6.2. Latency and Stopping Distance

Here, we evaluated the measured detection ranges with regard to the requirements of collision avoidance for industrial applications. Following the methodology used by Kim et al. [27], the minimum detection range was computed by summing up the distance the vehicle travels in the sensor-side latency and the total braking distance at speed v:

(21) $$d_{req}=vt_{L}+d_{b}(v)$$

where v is the speed of the vehicle in m/s, $t_{L}$ is the sensor-side latency in seconds, and $d_{b}(v)$ is the braking distance at speed v in meters. Equation (21) was computed for a velocity of 5 km/h because some companies prescribe this speed for indoor forklifts. Kim et al. reported that, in this case, a forklift truck driven on a dry concrete surface brakes within 2.2 m [27].

The sensor-side latency of the Firefly node is about 0.14 s, as shown in Table 10. The latency is defined as the time it takes to perform one ranging measurement after a person enters the field of view. Since the AMG8833 can provide 10 frames/s [33], it takes 100 ms at most to receive a new thermal frame. Then, 64 pixel values are sent using the 400 kHz I^2^C bus. Since each pixel has two bytes, the node waits for approximately 1200 bit periods (3.0 ms). Since the detection uses one frame, that is enough to make the final decision. The detection decisions reported in this paper were computed offline from the recorded frames. The two-gate detection, run on the Arduino Nano, took at most 5 ms per frame. Finally, the rangefinder boots (1.2 ms [97]) and performs one measurement (33 ms timing budget [65]).

The definition of the Gated-Fusion Efficiency is application-independent. We therefore show the resulting latency separately. The sensor stages in Table 10 describe a stationary node. Once it is integrated into a vehicle, the actuation delay of that vehicle adds to the required detection distance. For a speed of 5 km/h, the required detection distance is 2.4 m (Table 10). This lies within 5 m, the farthest tested distance at which F1 stayed above 0.75 for a standing person in all four environments, a margin of 2.6 m. A person holding a box was detected out to 4.2 m and a seated person out to 3.2 m, margins of 1.8 m and 0.8 m (Table 8).

### 6.3. Limitations

There are two sets of limitations to consider in this work: those that apply to the evaluation of the detector, and those that apply to this particular instantiation of the GFE. During the detector evaluation, the distance between the node and the subject varied from 1.2 to 7 m, only one human subject took part, only one frame was used to make a decision, and only four environments were considered. Therefore, these results can only be applied to situations with a similar setup. In three of the environments, the subject was wearing a T-shirt, while in the factory, the subject was wearing a hard hat and a high-visibility vest. In addition, in the factory, three postures were collected with three trials for each posture, but in the other environments, there was only one trial for the standing posture for each class and distance. Importantly, for all trials, the target and the sensor were stationary. However, this system is meant to be deployed on a mobile node. Therefore, the effect of the node’s motion is unknown. Thus, the results of this work are valid for a stationary node.

In addition, the negative class consisted only of a passive mannequin for the false-positive rate evaluation. This analysis did not include several possible causes of false positives such as running motors, heaters, operating machinery, exhausts, hot tooling, sunlit surfaces, and other sources of thermal clutter in motion. The effect these sources might have on detection rates or false-positive rates has not been tested. At this resolution, the sensor cannot differentiate between a human and another heat source of similar size and temperature. Some of these sources of false positives would cause the trigger to fire and wake the gated stage. There is a power cost for false wakes associated with drawing power in the gated stage, bounded by Equation (20).

A further limitation comes from the score itself, which bounds the scenes the detector can serve. It was computed using the temperature measured in the Celsius scale. Values less than 0 °C thus turn positive when squared, and therefore contrast within a cluster is not well captured near or below 0 °C. Hence, in environments near or below 0 °C, for instance, a cold-storage facility, the score should be computed using kelvin.

Four further limits apply to this instantiation of the GFE. The quality retained is a declaration, not a measurement. Based on the properties of the architecture, the parameter $\tilde{\rho }$ is set to 1 by construction. This simplifies the efficiency η to be equal to the power reduction Φ. As a result, the quality part of the figure of merit is not measured. The 18-cell experiment characterizes the thermal detector only. The quality of fused thermal-plus-ToF operation was not measured. Moreover, the input powers were taken as datasheet values for the given operating conditions. Therefore, efficiency was not a measurement on our implementation. The model also charges the gated stage steady-state draw only. Charging every triggered frame, the 1.2 ms boot of the rangefinder at its 45 mW continuous-ranging power lowers the efficiency at λ = 0.05 from 1.286 to 1.284. Efficiency is a curve as opposed to a single value. Our reported efficiency is a snapshot of this curve at a given operating point that depends on the use scenario.

## 7. Conclusions

We propose the Gated-Fusion Efficiency (GFE, η) as a figure of merit, to quantify the capability of triggered operation to exploit the trade-off between power and measurement quality offered by multi-sensor fusion with respect to the always-on scenario. We apply this metric to a testbed where a low-resolution thermal array acts as the trigger for a single-point time-of-flight rangefinder, for a stationary-human detection application. We derive two upper bounds on the potential power savings, namely, (i) one plus the power of the gated stage relative to the always-on root and (ii) behind a single trigger, and whatever the power of the gated stage, the inverse of the false-positive rate of the trigger. From this analysis, a guideline for triggered operation emerges: a trigger is suitable for a given gated stage if its false-positive rate is much smaller than the inverse of the relative power of the stage, meaning that more expensive stages require triggers with lower false-positive rates.

A total of 5267 frames were taken in four different environments at six different distances ranging from 1.2 m to 7 m. The mean F1-score for lab, room, and outdoors is 0.875. For the lab, an F1-score of 0.993 is obtained, with a score of 1.000 from 1.2 m to 3.2 m. While people can be detected in cluttered indoor and outdoor settings at distances of 5 m, performance decreases at the final range of 7 m. The factory setting was tested in three different postures and shows a decrease in performance at shorter ranges due to a decrease in viewable body area. For a person in a standing position, the F1-score is greater than 0.75 up to 5 m, though holding a box in front of the body decreases the range to 4.2 m, while for a person in a seated position the range is limited to 3.2 m. The point of drop-off in trigger detection depends on three factors: the width of the target, the minimum cluster size in the detector, and the angle each pixel covers. Given the width of the target and the minimum cluster size in the detector, the range in which performance decreases can be calculated for any resolution using the field of view specified in the datasheet and the number of pixels.

In all, our algorithm consists of two gates applied to different information sources. We use the contrast image for finding a cluster in order to localize the warm area. For the second gate, we use the raw temperature values instead of the residual. This avoids the self-confirmation of a residual-scored pipeline. One threshold serves all four environments. Leaving out test cells for threshold fitting causes a decrease in the average F1-score of 0.023 for residual- and of 0.008 for raw-scored detection when generalizing to these cells from the remaining cells of the three development environments. The detector has only one fitted parameter, the threshold, and does not use a temporal window. At a threshold of 0.30 dB, it indicates presence with a true-positive rate of 87.0% and produces 6.0% false alarms on mannequin sequences. Further experiments at the factory after choosing the threshold led to average F1-scores of 0.868 on sequences with a standing person.

## 8. Limitations and Future Work

For the given operating point and with datasheet powers, gating reduces the average power consumption for sensing. If we consider the power for sensor scope (excluding the 46 mW host), this gives rise to a reduction factor of 2.06× when a person is present in one frame out of every twenty. At the same occupancy, the reduction for the node scope is 1.29×. Task quality is not compromised since the rangefinder is used to sense objects that are detected by the trigger. Moreover, the reduction in power for node scope (1.29×) is close to the maximum possible reduction (1.33×); hence, further improvements in trigger quality would result in little improvement in power savings: the saving is hardware-capped rather than trigger-capped.

The next step is to carry GFE beyond the system where it was first deployed. The nearest candidates are perception stacks, where gating does cost quality. In mobile robotics and industrial vehicles, LiDAR is commonly fused with an optical camera. Here, the more expensive sensor provides more detail on objects that the less expensive sensor cannot resolve. For such a pairing, quality retention falls below one. GFE then arbitrates whether that sensor should be gated at all or left running continuously. Our refine-only combination avoided this decision. Future work includes three items. First, determining quality retention for fused tasks. Second, studying how η changes as scenes become denser and more complex. And finally, generalizing the caps for consecutive triggers. Our larger goal for sensing in Intelligent Manufacturing is to promote comparative reporting of the efficiency of triggered sensing solutions, including their declared scope of applicability and well-defined operating conditions, so that setups can be compared. This applies to triggered sensing for different combinations of modalities, e.g., thermal, acoustic, radar, or optical wake-up. This way, setups can be evaluated not just on their own, but also in comparison to alternative solutions.

Other directions include expanding the detection envelope to account for workers with different body sizes, workers wearing other clothing, protective equipment in settings other than the factory, and other orientations including crouching. Additional testing with multiple people in view would also be useful. Trials with active heat sources in the field of view, such as running motors, heaters, machinery, exhausts, hot tooling, sunlit surfaces, and moving warm objects, should measure how these sources change the detection and false-positive rates. Tests of the entire system should be conducted to determine the total delay from a person entering the scene to triggering of the stop signal. Validation on a moving platform is left as future work. Deployment can begin with stationary nodes, which the static trials reported here support. Later, the node could be affixed to an industrial vehicle approaching a worker to validate operation when the worker is standing or walking. Once validated in this manner, a system could be developed and placed on vehicles.:

## Figures and Tables

**Table 1:** AMG8833 and VL53L1X sensor specifications.

Parameter	AMG8833	VL53L1X
Sensor type	Thermopile array	Time-of-flight rangefinder
Resolution	8 × 8 pixels (64 elements)	Single point
Field of view	60° × 60° (7.5°/pixel)	27° (default region of interest, ROI)
Range	0 to 80 °C (object)	40 to 4000 mm
Quantization/accuracy	0.25 °C	±3% (calibrated)
NETD/timing budget	≈0.5 °C	33 ms per measurement
Frame rate	10 Hz	10 Hz (effective)
Output format	64 floats, row-major, °C	Distance in mm
Interface	I^2^C, 0 × 69, 400 kHz	I^2^C, 0 × 29, 400 kHz
Role in this paper	Detection (Gate 1 + Gate 2)	System distance/evaluation only

**Table 2:** Equipment used in data collection, with component models and roles in the detection and evaluation pipeline.

Equipment	Model	Quantity	Purpose
Thermal sensor	Panasonic AMG8833 (Adafruit breakout)	1	Primary detection sensor
ToF rangefinder	STMicroelectronics VL53L1X (Adafruit breakout)	1	System distance output
Microcontroller	Arduino Nano (ATmega328P, 16 MHz)	1	I^2^C polling + USB serial streaming
Mounting fixture	Tripod & breadboard	1	Horizontal-axis sensor mount
Tape measure	Standard	1	Ground-truth distance from tripod center to subject

**Table 3:** Range of pixel temperatures recorded by the AMG8833 in each experimental environment during data collection.

Temperature	Room	Outdoor	Lab	Factory
Min (°C)	18.5	−3.5	16.5	16.3
Max (°C)	26.3	16.3	25.3	25.3

**Table 4:** Frame count for test trials involving a human.

Environment	1.2 m	2.2 m	3.2 m	4.2 m	5 m	7 m
Lab	35	35	35	35	36	36
Room	19	49	36	59	36	36
Outdoor	37	37	36	36	35	35
Factory, standing	108	108	108	108	108	108
Factory, holding a box	108	108	108	108	108	108
Factory, seated	108	108	108	108	108	108

**Table 5:** Frame count for test trials involving mannequin (negative class).

Environment	1.2 m	2.2 m	3.2 m	4.2 m	5 m	7 m
Lab	32	37	55	78	52	51
Room	33	28	23	25	28	41
Outdoor	19	38	43	43	42	48
Factory, standing	108	108	108	108	108	108
Factory, holding a box	108	108	108	108	108	108
Factory, seated	108	108	108	108	108	108

**Table 6:** Per-cell mean ΔSNR (human–mannequin), in dB. The factory row (standing posture) is held-out data and is not among the 18 lab, room, and outdoor cells used for the sign and Wilcoxon tests.

Environment	1.2 m	2.2 m	3.2 m	4.2 m	5 m	7 m
Lab	+1.255	+0.872	+0.671	+0.576	+0.527	+0.514
Room	+0.878	+0.860	+0.595	+0.354	+0.390	−0.074
Outdoor	+0.423	+1.282	+2.025	+2.135	+1.565	+0.261
Factory (standing)	+1.236	+1.001	+0.925	+0.775	+0.628	+0.097

**Table 7:** Per-cell F1, standing human against standing mannequin, at the operating point $\tau^{*}$ = 0.30 dB (the F1 maximum on the 0.1 dB threshold grid).

Environment	1.2 m	2.2 m	3.2 m	4.2 m	5 m	7 m	Mean
Lab	1.000	1.000	1.000	0.986	0.986	0.986	0.993
Room	0.974	0.970	0.932	0.800	0.899	0.048	0.770
Outdoor	0.860	0.851	1.000	0.986	1.000	0.478	0.863
Factory	1.000	0.991	1.000	0.967	0.926	0.326	0.868

**Table 8:** Per-cell F1 in the factory environment for three postures, at $\tau^{*}$ = 0.30 dB.

Posture	1.2 m	2.2 m	3.2 m	4.2 m	5 m	7 m	Mean
Standing	1.000	0.991	1.000	0.967	0.926	0.326	0.868
Holding a box	1.000	1.000	1.000	0.941	0.633	0.088	0.777
Seated	1.000	0.976	0.761	0.018	0.000	0.018	0.462

**Table 9:** Φ and η are evaluated at λ=0.05 under different operating modes.

Operating Mode	$P_{B}$ (mW)	κ	Φ	η=Φ
ToF VL53L1X (10Hz/33ms time budget)	20	0.33	1.29×	1.29
ToF VL53L1X (continuous ranging)	~45	0.74	1.62×	1.62
Heavier compute stage	200	3.28	3.22×	3.22
Edge camera/vision	~2000	32.8	7.86×	7.86

**Table 10:** Computed latency and distance budget of the Firefly node at 5 km/h. Braking time assumes uniform deceleration.

	Frame Capture	Data Transfer	Detection	Rangefinder Boot	Ranging	Braking	Total
Time (s)	≤0.100	0.003	≤0.005	≤0.0012	0.033	3.17	≤3.31
Distance (m)	0.139	0.004	0.007	0.002	0.046	2.2	2.4

## Data Availability

The experimental data supporting the findings of this study are available in the Supplementary Materials.

## Supplementary Materials

The following supporting information can be downloaded at: https://www.mdpi.com/article/10.3390/s26206482/s1.
